# Iron and sulfate reduction structure microbial communities in (sub-)Antarctic sediments

**DOI:** 10.1038/s41396-021-01014-9

**Published:** 2021-06-21

**Authors:** Lea C. Wunder, David A. Aromokeye, Xiuran Yin, Tim Richter-Heitmann, Graciana Willis-Poratti, Annika Schnakenberg, Carolin Otersen, Ingrid Dohrmann, Miriam Römer, Gerhard Bohrmann, Sabine Kasten, Michael W. Friedrich

**Affiliations:** 1 Microbial Ecophysiology Group, Faculty of Biology/Chemistry, University of Bremen, Bremen, Germany; 2 Max Planck Institute for Marine Microbiology, Bremen, Germany; 3 MARUM – Center for Marine Environmental Sciences, University of Bremen, Bremen, Germany; 4 Instituto Antártico Argentino, Buenos Aires, Argentina; 5 Facultad de Ciencias Exactas, Universidad Nacional de La Plata, Buenos Aires, Argentina; 6 Alfred Wegener Institute Helmholtz Centre for Polar and Marine Research, Bremerhaven, Germany; 7 Faculty of Geosciences, University of Bremen, Bremen, Germany

**Keywords:** Microbial ecology, Biogeochemistry

## Abstract

Permanently cold marine sediments are heavily influenced by increased input of iron as a result of accelerated glacial melt, weathering, and erosion. The impact of such environmental changes on microbial communities in coastal sediments is poorly understood. We investigated geochemical parameters that shape microbial community compositions in anoxic surface sediments of four geochemically differing sites (Annenkov Trough, Church Trough, Cumberland Bay, Drygalski Trough) around South Georgia, Southern Ocean. Sulfate reduction prevails in Church Trough and iron reduction at the other sites, correlating with differing local microbial communities. Within the order *Desulfuromonadales*, the family Sva1033, not previously recognized for being capable of dissimilatory iron reduction, was detected at rather high relative abundances (up to 5%) while other members of *Desulfuromonadales* were less abundant (<0.6%). We propose that Sva1033 is capable of performing dissimilatory iron reduction in sediment incubations based on RNA stable isotope probing. Sulfate reducers, who maintain a high relative abundance of up to 30% of bacterial 16S rRNA genes at the iron reduction sites, were also active during iron reduction in the incubations. Thus, concurrent sulfate reduction is possibly masked by cryptic sulfur cycling, i.e., reoxidation or precipitation of produced sulfide at a small or undetectable pool size. Our results show the importance of iron and sulfate reduction, indicated by ferrous iron and sulfide, as processes that shape microbial communities and provide evidence for one of Sva1033’s metabolic capabilities in permanently cold marine sediments.

## Introduction

Organic matter degradation is the main source of electron donors and carbon for microbial metabolism in marine sediments [1, 2]. The estimated 5.39 × 10^29^ microbial cells in marine sediments [3] form a microbial food chain. Below the oxic zone, the anaerobic portion of this food chain starts with specialists, which perform hydrolytic and fermentative processes [4], and ends with anaerobically respiring microorganisms, which oxidize fermentation products with available terminal electron acceptors such as nitrate, Mn(IV), Fe(III), sulfate, and CO_2_. Because nitrate and Mn(IV) are rapidly depleted in the uppermost centimeters of most coastal and upper slope surface sediments [2, 5], sulfate and Fe(III) are the most abundant terminal electron acceptors utilized by microorganisms for mineralization of fermentation products in these depositional environments [6–8].

Iron enters the ocean from various sources including terrigenous origins via weathering and erosion and subsequent transport by rivers and windblown dust; hydrothermal vents [9]; melting sea ice and icebergs [10]; and glacial associated erosion, weathering, and meltwater [11–14]. Due to global warming, glacial associated input of iron is predicted to increase in the future, resulting in enhanced amounts of iron reaching coastal sediments and adjacent ocean areas especially in higher latitudes [13, 15, 16]. Sulfate, which is generally present in high concentrations in the water column (~28 mM), is supplied to the sediment by downward diffusion, accelerated by bio-irrigation and other advective processes [7, 8, 17]. In addition, it is the final product of reoxidation of sulfide [18, 19], which itself is produced by sulfate reduction [20], potentially resulting in a cryptic sulfur cycle [18, 19, 21]. Iron reduction is constrained by the reactivity and lower availability of ferric iron compared to sulfate [5, 22]. Therefore, while iron reduction is favored in certain marine settings [23, 24], organic matter oxidation by sulfate reduction is often more important than iron reduction in marine sediments [7, 8], a competition shown to be also regulated by the availability and reactivity of organic matter and ferric iron [22, 25].

Geochemical and biogeochemical factors have been previously shown to be key parameters shaping the microbial communities in marine sediments [1, 26, 27]. Besides the availability of terminal electron acceptors [1], i.e., Fe(III) and sulfate, these factors include quantity, composition, and reactivity of organic matter [26, 28, 29], sediment geochemistry [27, 30, 31], salinity [32], temperature [33], ocean currents [34], primary productivity in the overlying water column [35], and sedimentation rate [24].

The permanently cold surface sediments around the island of South Georgia in the South Atlantic Ocean, which we have investigated in this study, are influenced by high organic matter content around the shelf areas (0.65 wt% Cumberland Bay [36]), and high iron content within or close to the fjords (ref. [37], Cumberland Bay: total Fe solid phase 47 g/kg [38]; 0.7 wt% ferrihydrite and lepidocrocite [39]). In addition, the studied sediments were found to be characterized by widespread active methane seepage within the fjords and on the shelf, mostly associated with cross-shelf glacial troughs [36, 40, 41]. So far, we studied the microbial communities inhabiting deeper sediments (down to 10 m below seafloor) at three sites around South Georgia [42] and in the present study, a detailed analysis of the surface sediments at very fine scales is provided.

The sediments of the second study site Potter Cove, a small fjord located at the southwest of King George Island/Isla 25 de Mayo (South Shetland Islands) on the northern tip of the West Antarctic Peninsula, are characterized by a high input of iron from glacial meltwater and bedrock erosion [13, 14, 43]. Especially, sediments close to the glacier termination show a deeper ferruginous zone compared with sediments not directly influenced by glaciers [14] similarly to Cumberland Bay, South Georgia [41].

We hypothesize that differing geochemical characteristics in the surface sediments (top 20–30 cm) at various sites around South Georgia shape the local microbial communities. To test this hypothesis, four sites, located on the outer shelf (Annenkov Trough, Church Trough) or within or close to one of the fjords (Cumberland Bay, Drygalski Trough), were selected around the island of South Georgia. These sites were characterized by either high ferrous iron or hydrogen sulfide concentrations. The microbial communities of these sediments were investigated by 16S rRNA gene sequencing, quantitative PCR and RNA stable isotope probing (SIP) incubations. Correlation and multivariate regression analyses were performed to identify which geochemical parameters primarily shape the microbial community composition. The active iron-reducing microbial community of South Georgia sediments was compared to those in geochemically similar sediments of Potter Cove (Antarctic Peninsula) using RNA-SIP experiments.

## Materials and methods

### Study area and sampling

**Fig. 1:**
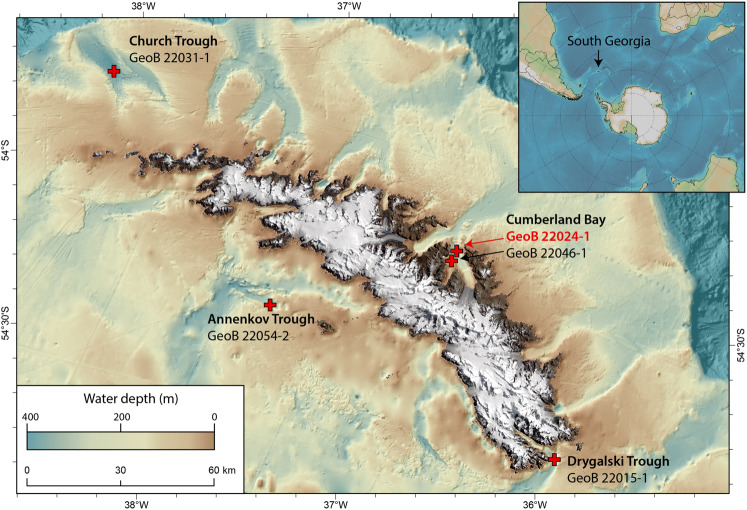
Sampling locations around South Georgia. Core identifications are displayed, the red marked core was used for SIP incubations.

Samples from South Georgia sediments were collected during the RV METEOR M134 expedition in January to February 2017 [41]. To study microbial communities in the surface sediments (Fig. 1), four sites were selected: two sites on the outer shelf (Church Trough, Annenkov Trough) and two sites within or close to one of the fjords (Cumberland Bay and Drygalski Trough, respectively). All sites are located close to (<500 m) areas where active methane seepage has been observed from the sediments during the M134 cruise [41]. Surface sediments were retrieved using a multicorer (MUC, length 50 cm). The exact coordinates and sampling information are provided in Table S1.

For each station, two replicate MUC cores were retrieved, one for pore water geochemistry and one for microbiology. Sampling for both geochemistry and microbiology was done on board of the ship in a cold room at 4 °C. Microbiology samples were frozen in liquid nitrogen and transported to Bremen for molecular biology analyses. In addition, a gravity core (10 m length) was retrieved from the site in Cumberland Bay (detailed information Table S1) and kept at 4 °C until and during transport back to Bremen where it was sectioned and stored anoxically until use.

Antarctic surface sediments from Potter Cove (King George Island/Isla 25 de Mayo), Station 13, were retrieved during a field campaign with a push core in January 2019 (detailed information Table S1). The sampling site (Fig. S1) and geochemistry were previously described in Henkel et al. [14].

### Pore water geochemistry

Pore water was retrieved from the MUC and gravity cores using rhizon samplers according to the procedure described by Seeberg-Elverfeldt et al. [44]. Dissolved iron(II) (Fe^2+^), phosphate (PO_4_^3−^), ammonium (NH_4_^+^), dissolved inorganic carbon (DIC) and silicate (SiO_2_) were measured on-board as described in Bohrmann et al. [41], while samples for sulfate (SO_4_^2−^, diluted 1:50 with Milli-Q water) and hydrogen sulfide (H_2_S, fixation in 2.5% zinc-acetate solution) measurements were stored for later analysis. H_2_S and SO_4_^2−^ measurements were performed following Oni et al. [45].

### DNA extraction and 16S rRNA gene sequencing

To explore the microbial communities in the South Georgia surface sediments, 1.5 g of frozen sediment was taken from 10 depths per site, selected according to the geochemical profiles, for DNA extraction. DNA extraction (by a phenol-chloroform protocol), PCR, and amplicon sequencing on a HiSeq 4000 System (Illumina, San Diego, CA; 2x 150 bp, only forward reads analyzed) at GATC GmbH (Konstanz, Germany) of bacterial and archaeal 16S rRNA genes were done following Aromokeye et al. [46]. The primer pair Bac515F (5′-GTGYCAGCMGCCGCGGTAA-3′; ref. [47]) and Bac805R (5′-GACTACHVGGGTATCTAATCC-3′; ref. [48]) targeted bacteria and the primer pair Arc519F (5′-CAGCMGCCGCGGTAA-3′; ref. [49]) and Arc806R (5′-GGACTACVSGGGTATCTAAT-3′; ref. [50]) targeted archaea. Unassigned reads or those assigned to chloroplasts, mitochondria, and archaea (in the bacterial dataset) or bacteria (in the archaeal dataset) were removed from the OTU tables. Normalization of sequencing data was done by calculating relative abundances, which were summed up for each taxon on all available ranks (for more details see supplementary methods, Table S2 and Fig. S2).

### SIP experiments using (sub-)Antarctic sediments

RNA-SIP incubations were set up in order to identify active iron reducers using the top sediments of a gravity core from Cumberland Bay, South Georgia (GeoB22024-1; 0–14 cm, stored at 4 °C). The setup is described in more detail in the supplementary methods. Briefly, 40 ml anoxic slurries were prepared at a ratio of 1:4 of sediment and sulfate-free artificial seawater (per liter 26.4 g NaCl, 11.2 g MgCl_2_·6 H_2_O, 1.5 g CaCl_2_·2 H_2_O, 0.7 g KCl, prepared with purified water (Milli-Q)). Before addition of substrates, slurries were pre-incubated at 5 °C in the dark for 4–6 days to allow for system equilibration and pre-reduce small amounts of alternative electron acceptors (e.g., nitrate) potentially present in the starting sediments. Four different treatments were set up in triplicates (Table S3), containing 0.5 mM ^13^C-labeled acetate as electron donor and carbon source and as electron acceptor either 5 mM lepidocrocite, 5 mM sulfate, or none. Lepidocrocite was chosen as easily reducible iron oxide typically found in surface sediments, including the study site [39]. One treatment contained 10 mM sodium molybdate in addition to lepidocrocite in order to inhibit sulfate reduction. For each treatment, a control with unlabeled (^12^C) acetate was set up. An unamended incubation with only sediment and artificial sea water was used as control. Aqueous Fe^2+^ formation was monitored during the course of the incubation for each bottle individually using a ferrozine assay [51], modified by fixing the samples in 0.5 M HCl in order to prevent further oxidation. Aqueous sulfate was measured at day 0 and day 15 (end point) of the incubation using a Metrohm 930 Compact IC Flex ion chromatograph. After 15 days, RNA was extracted from pooled triplicates in order to retrieve sufficient biomass for fractionation.

To support the observations from Cumberland Bay, a second SIP experiment was performed using Antarctic sediments from Potter Cove (push core 0–29 cm) with similar geochemistry as Cumberland Bay [14, 36]. For Potter Cove sediment incubations, the procedure for experimental setup and Fe^2+^ measurement was similar as described above with the modifications of using only 30 ml slurry (ratio 1:6) in 60- ml serum bottles, an incubation temperature of 2 °C and a pre-incubation time of 7 days. The single treatment contained 0.5 mM ^13^C-labeled or unlabeled acetate and 5 mM lepidocrocite as substrate. The incubation was carried out for 10 days.

### RNA-SIP

The steps of nucleic acid extraction, removal of DNA, quantification, density separation, and preparation of 16S rRNA sequencing were performed following a previously described protocol [52] with the following modifications. Briefly, nucleic acids were extracted from 15 ml slurry per treatment, using a phenol-chloroform extraction protocol, followed by DNase treatment and an additional phenol-chloroform purification and RNA precipitation step with isopropanol and sodium acetate. RNA was quantified with Quanti-iT RiboGreen and 1 µg was used for density separation by ultracentrifugation. This resulted in 14 fractions of which fraction 1 had the highest density (= heaviest) and fraction 14 the lowest. The RNA content of each fraction was quantified and fractions were defined and pooled by their RNA concentration-density profile as ultra-heavy = fraction 3 + 4 (1.814–1.826 g/ml); heavy = fraction 5 + 6 (1.799–1.810 g/ml); midpoint = fraction 7 + 8 (1.783–1.799 g/ml); light = fraction 9 + 10 (1.768–1.783 g/ml); ultra-light = fraction 11 + 12 (1.753–1.768 g/ml). The pooled fractions were used for cDNA synthesis. The bacterial 16S rRNA amplicon library was prepared as previously described [46] and paired-end sequenced at Novogene Co. Ltd. (Cambridge, UK) using Novaseq6000 platform (2x 250 bp). Sequencing analysis was done following Aromokeye et al. [46] with modifications described in the supplementary material (sequencing details Table S4 and Fig. S3) and in the 16S rRNA gene sequencing paragraph above.

### Quantitative PCR

Bacterial and archaeal 16S rRNA gene copy numbers of South Georgia surface sediments were determined by quantitative real-time PCR (qPCR). The qPCR assay followed Aromokeye et al. [53] with 1 ng DNA template and a cycling program of 95 °C: 5 min; 40 cycles at 95 °C: 15 or 30 s, 58 °C: 30 s, 72 °C: 40 s (Table S5); with efficiencies >80% and *R*^2^ > 0.99. For bacteria quantification, the primers Bac8Fmod (5′-AGAGTTTGATYMTGGCTCAG-3′; modified from ref. [54]) and Bac338Rmod (5′-GCWGCCWCCCGTAGGWGT-3′; modified from ref. [55]) were used. For archaea quantification Ar806F (5′-ATTAGATACCCSBGTAGTCC-3′; alternative name Arc787F in ref. [55]) and Ar912rt (5′-GTGCTCCCCCGCCAATTCCTTTA-3′; ref. [56]) were used. The gene copy number calculation was based on standard curves of 16S rRNA gene fragments of *Escherichia coli* (strain SB1) and *Methanosarcina barkeri* (strain DSM800), amplified with 27F (5′-AGAGTTTGATCCTGGCTCAG-3′; ref. [57]) and Ba1492 (5′-GGTTACCTTGTTACGACTT-3′; ref. [57]), and 109F (5′-ACKGCTCAGTAACACGT-3′; ref. [58]) and A1492 (5′-GGCTACCTTGTTACGACTT-3′; ref. [57]) primer pairs, respectively (Table S5), prepared and analyzed according to Reyes et al. [59].

In surface sediment samples and Cumberland Bay SIP fractions, the functional gene for sulfate reduction, alpha-subunit of the dissimilatory sulfite reductase (*dsrA*), was used for the quantification of sulfate reducers following the qPCR protocol of Reyes et al. [59]. For the qPCR reaction, the primer pair DSR1-F+ (5′-ACSCACTGGAAGCACGGCGG-3′; ref. [60]) and DSR-R (5′-GTGGMRCCGTGCAKRTTGG-3′; ref. [60]) was used. As standard, the *dsrAB* gene of *Desulfovibrio burkinensis* (strain DSM 6830) was amplified with a mix of modified DSR1F/DSR4R primers (for details see Reyes et al. [59]).

### Statistical analysis

Selected pairwise Pearson correlations were calculated between OTU abundances, gene copy numbers, and environmental variables. A distance-based redundancy analysis (dbRDA) was performed on a Bray-Curtis dissimilarity distance matrix between geochemical parameters and bacterial relative abundances from sequencing and tested for predictor variable collinearity, statistical significance (at *p* < 0.05) for the full model, and constrains for each variable. *P* values were adjusted for multiple testing according to the false discovery method [61], if necessary. All statistical analysis and figures were made within the R environment version 3.6.1 [62] using the vegan package [63].

Closest sequences of the most abundant OTUs assigned as Sva1033 were exported from 16S rRNA gene ARB tree of Silva release 138 (SILVA_138_SSURef_NR99_05_01_20, ref. [64]; >1300 bp, randomly selected) as well as closest named neighboring clusters. A maximum-likelihood tree was inferred with RAxML (version 8.2.11, ref. [65]) using the GTRGAMMA model with 1000 times rapid bootstrapping. The tree file was visualized using iTOL software (v4, ref. [66]) and edited in Inkscape (version 1.0.1, ref. [67]).

## Results

### Pore water geochemistry

**Fig. 2:**
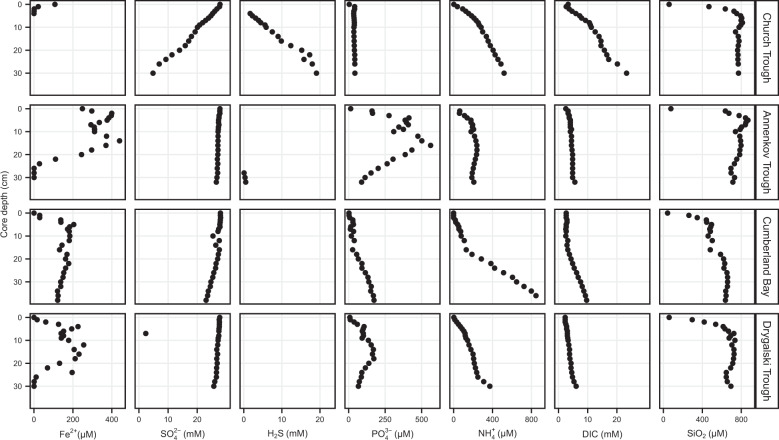
Pore water concentrations of iron(II) (Fe^2+^), sulfate (SO_4_^2‒^), sulfide (H_2_S), phosphate (PO_4_^3‒^), ammonium (NH_4_^+^), dissolved inorganic carbon (DIC), and silicate (SiO_2_) in surface sediments, South Georgia. All missing values in Fe^2+^ and H_2_S profiles were for data points below detection limit.

Seven different geochemical pore water parameters were analyzed in the context of their correlation to the microbial community in the sediment. Notable differences across the sites were observed in the pore water concentrations and profiles of Fe^2+^, SO_4_^2−^, and H_2_S (Fig. 2). Of all parameters, Fe^2+^ concentrations showed the strongest variability among the study sites.

At the sampling site in Church Trough, Fe^2+^ became rapidly depleted with depth and undetectable below 3 cm core depth. Below this depth, downward increasing H_2_S concentrations (up to 20 mM at 30 cm) coincided with decreasing SO_4_^2−^ concentrations (28 mM at 0 cm to 5 mM at 30 cm). This defines the sampling site in Church Trough as being sulfide-rich (Fig. 2). At the other sampling sites, Fe^2+^ predominated in the sampled sediment interval. The maximum Fe^2+^ concentration was measured in the sediments sampled in Annenkov Trough (440 µM). Therein, Fe^2+^ concentrations became completely depleted down-core followed by detection of low H_2_S concentrations below 30 cm (500 µM). In the investigated sediments of both Cumberland Bay and Drygalski Trough, Fe^2+^ was detected throughout the sampled sediment depth with maximum concentrations of 204 and 256 µM, respectively, while H_2_S was below detection limit. The predominance of Fe^2+^ over H_2_S in the sediments of these sites thus defines them as iron-rich sites (Fig. 2). In the sediments at the iron-rich sites, SO_4_^2−^ concentrations stayed stable with depth (~28 mM) with only minor decreases observed in the surface sediments collected in Cumberland Bay (below 18 cm from 27 to 23 mM).

Profiles of NH_4_^+^ and DIC showed similar distribution and shapes with increasing values over depth at all sites (Fig. 2). DIC concentrations in Church Trough sediments reached double the concentrations observed in the sediments of the other sites toward the bottom of the core. Close to the surface, SiO_2_ concentrations increased downward rapidly to a maximum value differing between sites and stayed stable through the rest of the core.

### Microbial community composition and abundance estimation

**Fig. 3:**
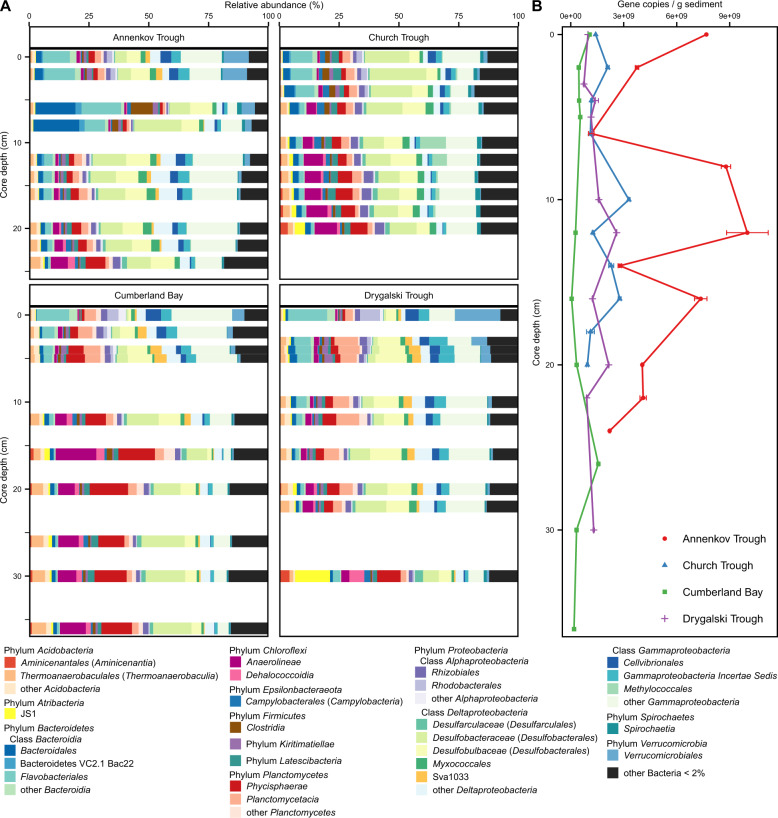
Bacterial community composition and gene copy numbers in South Georgia surface sediments. **A** Relative abundance of bacterial 16S rRNA genes in 10 depths of Annenkov Trough, Church Trough, Cumberland Bay and Drygalski Trough. **B** Bacterial 16S rRNA gene copies per gram wet sediment of samples displayed in (**A**) with error bars displaying SD of technical qPCR replicates.

The bacterial community composition of South Georgia surface sediments was investigated by 16S rRNA gene sequencing. Distinct similarities were observed in the distribution of core communities across all sites (Fig. 3A). Relative abundance of sequences falling into *Flavobacteriales*, the *Alphaproteobacteria Rhodobacterales* (mostly *Rhodobacteraceae*); the *Gammaproteobacteria Cellvibrionales* (mostly *Halieaceae*); *Planctomycetacia* (mostly *Pirellulaceae*); and *Verrucomicrobiales* (mostly *Rubritaleaceae*) decreased with sediment depth, while the relative abundance of sequences associated with *Anaerolineae* (phylum *Chloroflexi*), *Phycisphaerae* (mostly clade MSBL9), and the *Atribacteria* JS1 increased.

Certain differences between sites were also evident: the relative abundance of *Desulfobacteraceae* was low (<5%) in the top sediments of the iron-rich sites in Annenkov Trough, Cumberland Bay, and Drygalski Trough and only increased down-core, but was very high (18%) in the top sediments of the sulfide-rich site Church Trough. One major difference between all sites was the presence of *Methylococcales* throughout the Church Trough core (up to 11%), whereas it was present in very low relative abundance at the other sites (<0.5%). Conversely, the *Desulfuromonadales* family Sva1033 was present in all sites but with very low  abundance in the Church Trough core (5% vs. 0.6%).

Bacterial 16S rRNA genes were quantified by qPCR with gene copy numbers ranging between 5 × 10^7^ and 1 × 10^10^ copies per gram sediment and differed significantly between sites (Fig. 3B). Specifically, highest gene copy number estimates were obtained from the sediments sampled in Annenkov Trough (up to 1 × 10^10^ at 12 cm) and lowest in Cumberland Bay (5 × 10^7^–1.6 × 10^9^).

Archaeal sequences recovered after quality filtering were much less compared to bacterial sequences (Table S2). A complete depth profile of the archaeal community was only possible for samples derived from Church Trough and Cumberland Bay (Fig. S4A), because the archaeal read numbers and sequencing depth from the majority of the sampled depths in Drygalski Trough and Annenkov Trough were too low (<900 reads, Table S2). The most abundant archaea in all sites were *Bathyarchaeota* (up to 31% in Cumberland Bay) and the genus *Candidatus* “Nitrosopumilus” (up to 70% in Cumberland Bay) with their relative abundance decreasing with depth (Fig. S4A). In Church Trough sediments, anaerobic methane oxidizing archaea groups ANME-2a-2b and -2c were found in high relative abundances below 5 cm core depth (ANME-2a-2b up to 15%, ANME-2c up to 31%). Archaeal 16S rRNA gene copy numbers from qPCR were in general a magnitude lower than the bacterial gene copies (Fig. S4B). In contrast to bacterial copy numbers, archaeal copy numbers were highest in Church Trough sediments ranging from 8.6 × 10^7^ to 4.6 × 10^8^ copies per gram sediment. Again, the lowest gene copy numbers of archaea were detected in samples from Cumberland Bay with only 1.6 × 10^7^ to 1.1 × 10^8^ copies.

### Statistical correlations between geochemical parameters and bacterial communities

**Fig. 4:**
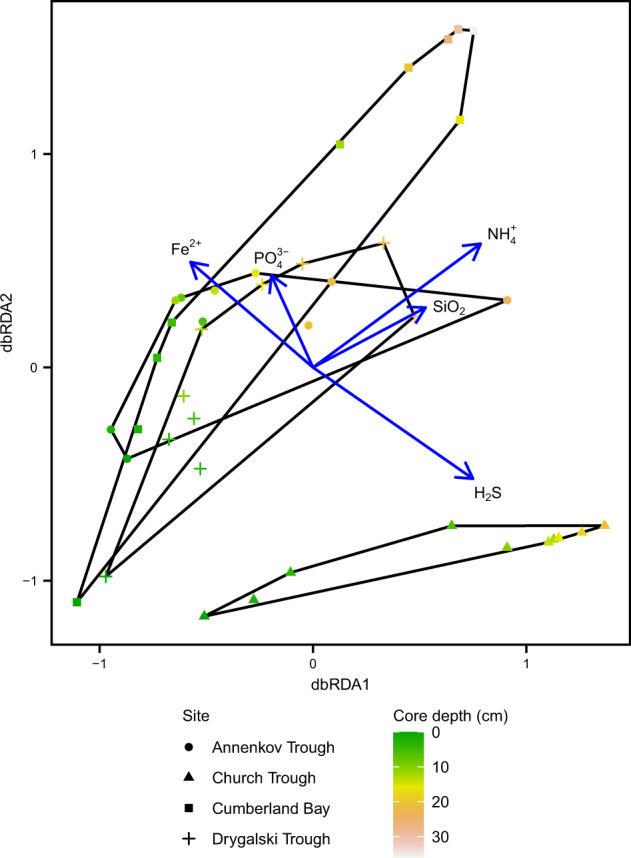
Distance-based redundancy analysis (dbRDA) ordination plot of bacterial communities in surface sediments of South Georgia. Sample points are distinguished by site and core depth by shape and color, respectively. dbRDA1 (variation 47%) and dbRDA2 (variation 22%) axes are displayed, which constrain the Bray-Curtis distance matrix with geochemical parameters Fe^2+^, PO_4_^3−^, NH_4_^+^, SiO_2_, and H_2_S. The total model (*F* = 4.99, *p* < 0.01, Df: 5, 34) and each individual parameter (*p* < 0.05) was significant.

In order to identify potential geochemical filters that shape the microbial communities across all sites, a dbRDA was performed (Fig. 4, *F* = 4.99, *p* < 0.01, Df: 5, 34). Fe^2+^, PO_4_^3−^, NH_4_^+^, SiO_2_, and H_2_S were included as explanatory variables in the model and together explained 42% of the total variation in the bacterial community. DIC and SO_4_^2^^−^ were removed due to collinearity to other factors (NH_4_^+^ and H_2_S, respectively). Increasing NH_4_^+^ concentrations with sediment depth explained most of the variation of the bacterial community (*F* = 5.85, *p* < 0.01), followed by H_2_S (*F* = 3.61, *p* < 0.01), SiO_2_ (*F* = 2.90, *p* < 0.01), Fe^2+^ (*F* = 2.25, *p* = 0.012), and PO_4_^3−^ (*F* = 2.12, *p* = 0.015). The clustering in the site ordination space showed a clear distinction between Church Trough and the other three sites (Fig. 4). The model strongly attributed this distinction to Fe^2+^ and H_2_S concentration differences between sites. Accordingly, removing these two variables from the model caused the clustering by sampling site in the ordination to disappear (Fig. S5).

**Fig. 5:**
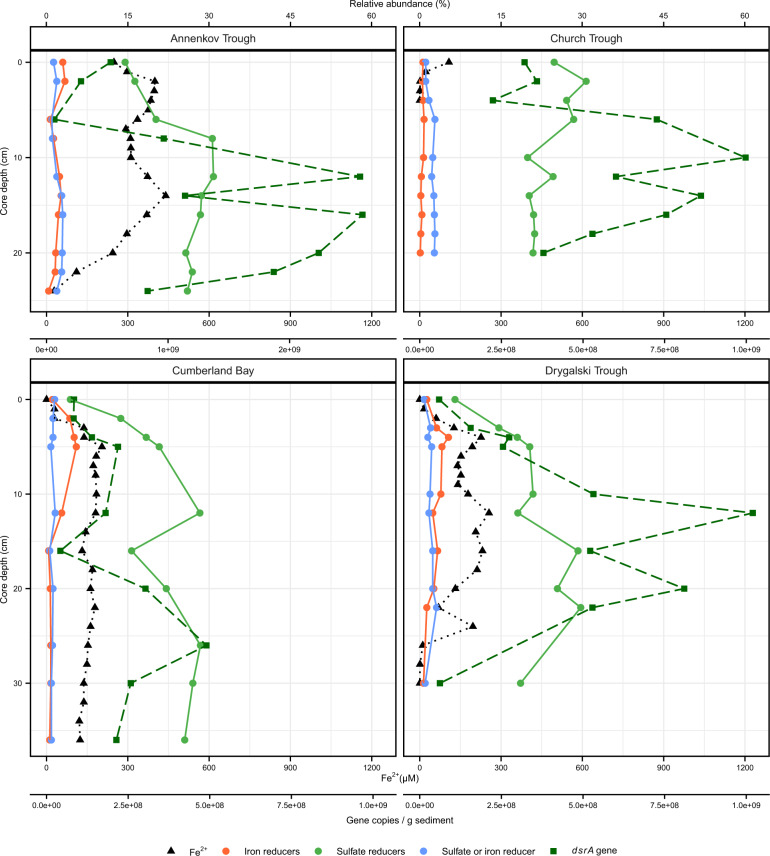
Depth profile of contribution of sulfate and iron reducing microorganisms in *Deltaproteobacteria* to bacterial community and quantification of sulfate reducers (*dsr*A gene) in South Georgia surface sediments. Relative abundance of 16S rRNA gene of taxa known for iron and/or sulfate reducing capabilities within *Deltaproteobacteria* (details in the text) was summed up per sediment depth. Fe^2+^ profile from Fig. 2 and *dsrA* gene copies per gram wet sediment  are displayed. Note the different scale for gene copies/g sediment for Annenkov Trough. Sequences of taxa known for iron reducing capabilities consisted of >78% Sva1033 in all depths of Annenkov Trough, Cumberland Bay, and Drygalski Trough.

Since H_2_S and Fe^2+^ concentrations, as indicators for sulfate and iron reduction, were identified as the key environmental factors for the microbial community composition in the sediments (Fig. 4), correlations between these geochemical parameters and taxa known to possess the capability of sulfate and iron reduction were performed. Therefore, relative abundance of known sulfate reducers within the *Deltaproteobacteria* was summed up for each sample (Fig. 5). This included the taxa *Desulfarculales* [68], *Desulfobacterales* [69], *Desulfohalobiaceae* [70], clade NB1-j [71], clade SAR324 [72], clade Sva0485 [71], and *Syntrophobacterales* [73]. Among the known iron reducers from marine sediments (e.g., [74–79]), members of *Desulfuromonadales* were the most abundant clade in this study. Summed up individual families included *Desulfuromonadaceae* [75, 80], *Geobacteraceae* [74] and *Deferrisoma* [81], and the family Sva1033 for which iron reducing capabilities were recently suggested [82]. The taxa *Desulfovibrionaceae* [83, 84] and *Myxococcales* [85] were separated as taxa known for both sulfate and iron reduction.

The relative abundance of sulfate reducers was high across all sites (8–30%; Fig. 5), despite the absence of indication for sulfate reduction, i.e., sulfide accumulation, in the pore water of Cumberland Bay, Drygalski Trough, and Annenkov Trough (Fig. 2). The presence of organisms capable of sulfate reduction was confirmed by qPCR of the *dsrA* gene as a functional marker gene for sulfate reduction: across all sites, the *dsrA* gene copies varied between 4.2 × 10^7^ (Cumberland Bay) and 2.6 × 10^9^ copies per gram sediment (Annenkov Trough; Fig. 5) and were positively correlated to the bacterial gene copies (*r* = 0.74, *p* < 0.001). Compared to sulfate reducers, the relative abundance of known iron reducers was lower across the iron-rich sites (1–5%; Fig. 5). In Church Trough sediments, where sulfate reduction was apparent, relative abundance of iron reducers was lower compared to the other sites (<0.6%). A significant correlation between relative abundance of iron reducers and Fe^2+^ concentrations was found for Drygalski Trough (*r* = 0.77, *p* < 0.01). Correlations were also calculated for the most dominant members of the order *Desulfuromonadales*, the family Sva1033 (Figs. 3 and S6). Sva1033 showed significant correlations with Fe^2+^ for Annenkov Trough (*r* = 0.63, *p* = 0.049) and Drygalski Trough (*r* = 0.77, *p* < 0.01), but not Cumberland Bay. Although the highest relative abundance of known (or proposed) bacteria capable of iron reduction was found in Cumberland Bay sediments, depth-wise relative distribution of iron reducers did not correlate with Fe^2+^ concentrations. Instead, a correlation was found between Fe^2+^ concentration and relative abundance of known sulfate reducers (*r* = 0.78, *p* < 0.01).

### SIP experiments with (sub-)Antarctic sediments

The abundance and distribution of known iron and sulfate reducers in the sediment communities raised questions about their metabolic activities in this environment. Sva1033 was the dominant member of *Desulfuromonadales* (Fig. S6), an order known for its iron reducing capabilities (e.g., [74–79]). It was found across all iron-rich sites, but this clade is only so far predicted—but not proven—to perform iron reduction due to phylogenetic affiliation to *Desulfuromonadales* (ref. [82], Fig. S7). Counterintuitively, sulfate reducers were significantly more abundant compared to iron reducers in the sites where iron reduction prevailed (Fig. 5). To further investigate these observations, we set up RNA-SIP incubations using acetate as ^13^C-labeled substrate with Cumberland Bay surface sediments in order to label active acetate oxidizers with the prediction that iron-reducing microorganisms capable of utilizing acetate as electron donor will be labeled in the heavy fractions [78, 86]. Given that obtaining a pure culture for Sva1033 was outside the scope of this study, we aimed with this strategy to obtain an indirect indication for iron reduction capability in the Sva1033 clade. In these incubations, increasing Fe^2+^ concentrations were detected in all treatments without significant differences between them, except in the treatment with acetate, lepidocrocite, and molybdate (Fig. S8A). In the molybdate-amended treatment, only moderate increase in Fe^2+^ concentrations was observed over time. No further increase of Fe^2+^ concentrations was detected by day 15 of the incubation experiment in all treatments. Sulfate was measured over time (Fig. S8B). A general trend of changing sulfate concentrations could only be observed in the acetate only treatment (decrease by up to 0.4 mM). Meanwhile, the range of sulfate concentration was different in the treatment amended with 5 mM sulfate (3.8–5.7 mM).

**Fig. 6:**
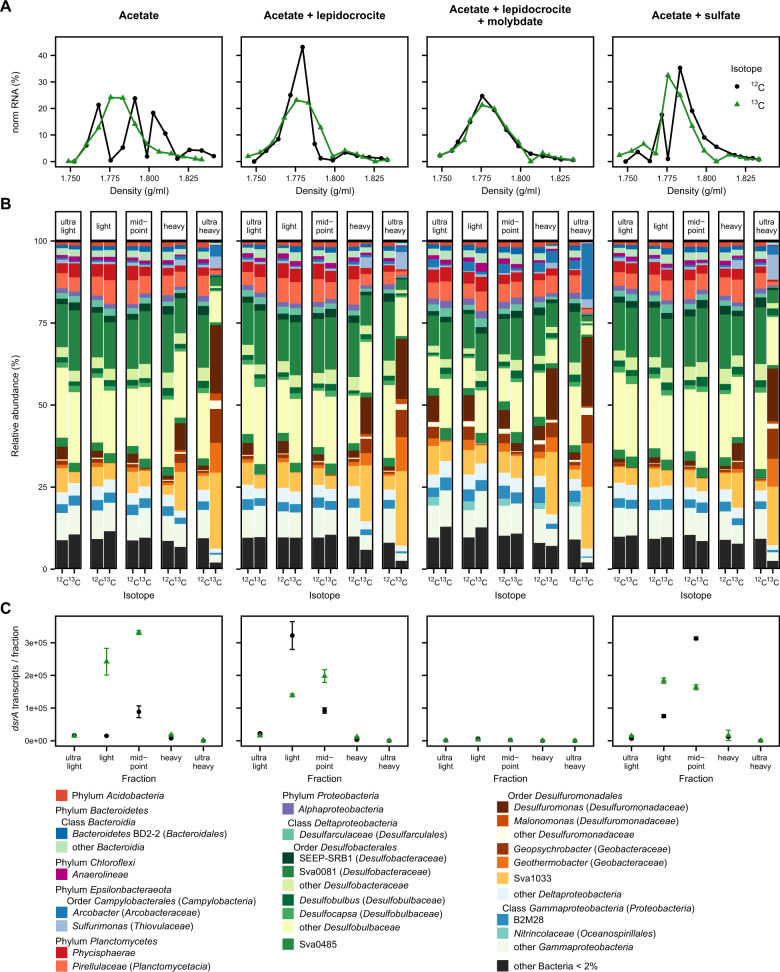
Results of SIP incubation with Cumberland Bay sediment. **A** Visualization of RNA density separation with normalized RNA amount (ng RNA in fraction per total recovered ng RNA of sample). **B** Density separated 16S rRNA community composition. **C** *dsrA* transcript copies per ng cDNA from recovered RNA per fraction with SD of technical qPCR replicates as error bars. In the molybdate-amended treatment, the transcript copies were below detection limit (<100 copies) for the ultra-heavy fraction and highest in the light fraction with 3000 (^13^C) to 6700 (^12^C) copies per ng cDNA from recovered RNA. Legend of **A** corresponds to **C**.

*Deltaproteobacteria* dominated the general bacterial community in the five defined gradient fractions per treatment (ultra-light, light, midpoint, heavy, ultra-heavy) after isopycnic separation. Their relative abundance ranged from at least 50% up to over 80% in some ^13^C ultra-heavy fractions. Clear differences were observed between the communities in the light and heavy fractions, thus confirming that the SIP separation was successful (Fig. 6A, B). The mostly enriched taxon in the ^13^C heavy and ultra-heavy fractions of all treatments was *Desulfuromonadales* within the *Deltaproteobacteria* (yellow-orange-brown in Fig. 6B), including the family Sva1033 (10–23%), *Desulfuromonas* (8–21%), *Geopsychrobacter* (8–10%), and *Geothermobacter* (8–13%). In total, the order *Desulfuromonadales* was more abundant in the ^13^C incubations, reaching up to 70% in the ultra-heavy fractions, compared to the ^12^C control incubations where their relative abundance was below 25% and mostly in the lighter fractions. Their relative abundance was slightly lower in the ultra-heavy fractions of the sulfate amended treatments (55%) compared to the other treatments (64–69%).

Within the class *Deltaproteobacteria*, the other abundant taxa were members of *Desulfobacterales*: *Desulfobacteraceae*, especially clade Sva0081, and *Desulfobulbaceae*, which together reached abundances of up to 50%. These groups of known sulfate reducers were more abundant in the ^13^C acetate and sulfate amended ultra-heavy fraction (23%) compared to the other ^13^C acetate amended treatments (6–18%). The lowest relative proportion of sulfate reducers in the ultra-heavy fraction (6%) was observed in the molybdate amended treatment. Quantification of the *dsrA* transcripts (Fig. 6C) showed very low copy numbers in the control treatment with molybdate (0–6700 transcript copies/ng cDNA from recovered RNA per fraction) compared to the other treatments (up to 300 000 transcript copies/ng cDNA per fraction). Sulfur oxidizing bacteria, *Arcobacter* and *Sulfurimonas* [87, 88] were the other enriched taxa in the ultra-heavy fractions. *Arcobacter* was mainly enriched in the ^13^C acetate, lepidocrocite, and molybdate treatment (17%) while *Sulfurimonas* was enriched in the treatments with acetate and lepidocrocite or sulfate (5–8%).

**Fig. 7:**
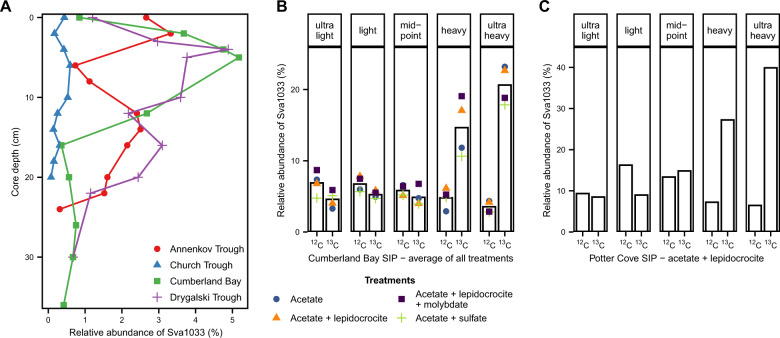
Abundance and activity of Sva1033. Relative abundance of Sva1033 in South Georgia surface sediments (**A**) and in SIP incubations with Cumberland Bay (**B**) and Potter Cove sediment (**C**). **A** Separation between sites by color and shape. **B** Averaged relative abundance of Sva1033 across all SIP treatments displayed by bars while relative abundance of each treatment is individually displayed by data points distinguishable by shape and color.

In addition to SIP incubations with surface sediments from Cumberland Bay, a SIP treatment amended with acetate and lepidocrocite was set up with sediments from Potter Cove, Antarctica, as a geochemically similar site in order to compare iron reducing communities from different locations. SIP was performed after running the incubation for 10 days from which iron reduction was observed (Fig. S8C). The most dominant enriched taxon in the ^13^C ultra-heavy fraction was the family Sva1033 (40% of bacterial 16S rRNA genes; Figs. 7C and S9), a similar observation to the SIP incubations with sediments from Cumberland Bay.

In order to investigate the phylogenetic relations of the most-enriched taxon Sva1033 (Fig. 7), a phylogenetic tree was constructed with the closest neighbors to the most abundant OTUs assigned as Sva1033 in South Georgia in situ sediments and Cumberland Bay SIP incubations and the closest neighboring clades from the Silva ARB tree (release 138). OTUs detected in situ were closely related to OTUs detected in the SIP experiment (Fig. S7). The clade closest related to the family Sva1033 was “Desulfuromonas 2” (as assigned by Silva 138).

## Discussion

Permanently cold coastal sediments from sub-Antarctic and Antarctic regions are subject to increased input of iron and other terrigenous compounds as a consequence of intensified weathering, erosion, and glacial melt due to observed global warming [10–15, 89, 90]. The impact of these altered element and material flux on the microbial communities in such sediments is currently understudied. Likewise, the bacterial communities present in surface sediments around the sub-Antarctic island South Georgia were not previously studied in detail. This study investigated the impact of environmental change on microbial communities in permanently cold (sub-)Antarctic sediments. Our findings show how geochemical characteristics such as the predominant electron accepting process and quality of organic matter potentially shape sediment communities in various sites around South Georgia. Importantly, in the iron reduction sites, we obtained evidence for dissimilatory iron reduction as one of Sva1033 clade’s ecological roles in permanently cold sediments using RNA-SIP. Finally, indications for concurrent sulfate reduction were obtained, despite the dominance of iron reduction in incubation experiments.

### Geochemical footprints shape microbial community composition

Selective survival of taxa buried below the upper 10 cm bioturbation zone has been identified as the significant process relevant for microbial community assembly in marine sediments [91–94]. Using the geochemical parameters as environmental factors for selection of the microbial community composition in the dbRDA (Figs. 4 and S5), various trends were observed. For example, depth-wise variation in community composition across all sites was strongly explained by the ammonium concentrations (Fig. 4), whose presence—along with DIC—is an indicator for organic matter degradation [95]. This was reflected in the core microbial community; the taxa *Flavobacteriales*, *Rhodobacterales*, *Cellvibrionales*, and *Verrucomicrobiales*, known for degradation of labile organic matter such as proteins, amino acids, polysaccharides, and simple sugars [96–99], were more abundant in the surface and decreased with depth across all sites (Fig. 3A). A similar trend was previously observed for some of these taxa in sediments of the Antarctic shelf [100]. In contrast, known “persister” microorganisms [91–93] such as *Anaerolineae*, *Phycisphaerae*, and the *Atribacteria* clade JS1 [101–104], showed a consistent increase in relative abundance along increasing depth across all sites (Fig. 3A). Differing supply of fresh organic matter on the outer shelf sites (Church Trough and Annenkov Trough) compared to sites located closer to the island was a possible explanation for likely higher microbial activity at these sites, as corroborating data from the geochemical profiles and gene copy numbers of microorganisms in the sediments (Figs. 2, 3B, 5, and S4B) indicated. This idea was supported by known large phytoplankton blooms and high primary production on the outer shelves around South Georgia [37, 40, 105].

Beyond ammonium shaping the communities along the sediment depth gradient, the dbRDA similarly showed a distinct selection of microbial communities in the study sites based on ferrous iron and sulfide concentrations. Thus, the likely dominant TEAP, i.e., iron and sulfate reduction, served as a factor for identifying the sites as either a group of iron reduction sites (Annenkov Trough, Cumberland Bay, Drygalski Trough) or sulfate reduction site (Church Trough; Fig. 4). A strong dependency of microbial community composition on TEAP was previously demonstrated in deeper sediments (down to 10 m below seafloor) from South Georgia [42] and from the Baffin Bay in the Arctic [27], in which iron and sulfate or iron and manganese reduction dominated, respectively.

Since ferrous iron and sulfide as products of microbial iron and sulfate reduction, respectively, were recognized by dbRDA as the environmental factors in our sediments to shape local communities, we hypothesize that the microorganisms contributing to these processes are important members of the microbial community. Thus identification of potential sulfate and iron reducers in the sediments will reveal which microorganisms are likely involved in the terminal respiratory processes. Amongst the sulfate reducers (Fig. 5), *Desulfobacteraceae*, *Desulfobulbaceae*, and *Desulfarculaceae* were most dominant, even down to the deeper layers of Cumberland Bay and Church Trough sediments [42]. The order *Desulfuromonadales* harbors many species with the metabolic capability to perform dissimilatory iron reduction [74, 75, 77], but also sulfur reduction [106–110] and even a few microorganisms are capable of sulfate reduction [111]. Members of *Desulfuromonadales* are typically found in ferruginous sediments (e.g., [45, 112, 113]). This order was the most abundant potential dissimilatory iron reducing clade in this study. Here, the main representative identified was the family Sva1033 (Figs. 3A, 7A, and S6), which was recently suggested to be capable of iron reduction in a Terrestrial Mud Volcano site [82] and Arctic sediments [112]. Based on the calculated phylogenetic tree (Fig. S7), this family is closely related to the clade “Desulfuromonas 2” (as assigned by Silva release 138 [64]). Until now, there are no cultivated members of this clade and its metabolic capabilities are yet to be confirmed. The significant correlation between depth-wise Fe^2+^ concentrations with relative abundance of Sva1033 in Annenkov Trough and Drygalski Trough (Fig. S6) strengthens the hypothesis that Sva1033 is involved in microbial iron reduction in surface sediments of South Georgia.

### The Sva1033 clade is capable of dissimilatory iron reduction

As the Sva1033 clade was first identified in Arctic sediments [114], we tested the hypothesis that this clade is ecologically adapted to perform iron reduction as one of its metabolic capabilities in permanently cold sediments. This was done by setting up RNA-SIP incubations using acetate as labeled substrate with Cumberland Bay and Antarctic Potter Cove sediments, especially as both Potter Cove and Cumberland Bay are characterized by a broad ferruginous zone [14]. Due to thermodynamic constraints in dissimilatory utilization, acetate has been frequently used successfully for specifically tracing anaerobically respiring microorganisms such as iron reducers (e.g., [78, 86]). Within Cumberland Bay sediments, iron reduction is very likely the dominant TEAP occurring in all SIP incubations as indicated by the increasing Fe^2+^ concentrations in the treatments, including controls (Fig. S8A). The slurry likely retained endogenous iron oxides and organic matter from the original sediment. In surface sediments from Cumberland Bay (same sampling site but previous expedition), total Fe content of the solid phase of 46.8 g/kg was reported [38], of which ferrihydrite and lepidocrocite contributed 0.65–0.7 wt% Fe [39]. Therefore, iron reduction could be stimulated without the amendment of additional electron acceptors or donors (see unamended control treatment, Fig. S8A). Given the similarity in the microbial community composition and proportion of enriched taxa in the heavy fractions (Fig. 6B), we conclude that dissimilatory iron reduction is the most likely dominant process conducted by the labeled taxa enriched in the heavy fractions of all treatments, i.e., members of *Desulfuromonadales* with Sva1033 as the most abundant taxon. This conclusion was supported by the observed similar geochemistry in the treatments (Fig. S8). The possibility that other processes such as sulfate and sulfur reduction, which could occur in these incubations, stimulated the enrichment of *Desulfuromonadales* is not supported by the formation of Fe^2+^ in the incubation experiment over time (Fig. S8A). The likelihood that Sva1033 performs iron reduction in situ as in the RNA-SIP incubations is supported by the close relation of OTU sequences from the in situ sediment and the SIP experiment (Fig. S7). Likewise, in Potter Cove sediment incubations, Sva1033 was also identified as the most dominant organism taking up the acetate label (40% relative abundance in ultra-heavy fraction, Figs. 7C and S9). Our study thus provides evidence for the capability for microbial iron reduction in the uncultured Sva1033 clade from permanently cold (sub-)Antarctic sediments with acetate as electron donor and carbon substrate (Fig. 7). Other taxa enriched in the heavy fractions of the SIP experiments included *Desulfuromonas*, *Geopsychrobacter*, and *Geothermobacter*, species with known iron reducing capabilities [75, 80, 115, 116].

### Activity of sulfate reducers in the iron reduction sites

Sulfate reducers are metabolically flexible. Their primary metabolic capabilities are essential for the global sulfur cycle as utilizers of the most oxidized form of sulfur [117] and they are capable of syntrophic growth, e.g., with methanogens [118, 119]. In addition, sulfate reducers are capable of growth with TEAPs such as nitrate [84, 120] and Fe(III) under sulfate limitation [83, 121]. In similar permanently cold marine sediments from the Arctic, sulfate reducers were relatively less abundant compared to iron reducers when iron reduction predominated [27, 112], which is in contrast to this study (Fig. 5). Despite the high abundance of sulfate reducers in the sediments of the iron-rich sites (Figs. 3 and 5), evidence for sulfate reduction was not directly obtained from the pore water profiles (Fig. 2). Hence, an open question emerges regarding the metabolism that keeps sulfate reducers persistent across these sites such that their relative abundances outnumber the iron reducers who likely perform the clearly more dominant TEAP in situ (Fig. 2). We hypothesize for our study that sulfate reduction, masked by the reoxidation of the produced sulfide back to sulfate, fuels the persistence of sulfate reducers [19, 21] in the iron reduction sites.

The results from SIP incubations showed that sulfate reducers were present and active in all treatments. However, they were present in higher abundance in the light compared to the heavy fractions (Fig. 6B). This can be explained by their high abundance in the starting sediment material (42%, Fig. S10). Sulfate reducers responded to the addition of electron acceptor as evidenced by their increased relative abundance in the heavy fractions of the sulfate-amended treatment compared to the other treatments (25% vs. 7–18% ultra-heavy labeled fraction). In general, the lower enrichment of sulfate reducers in the heavy fractions compared to the potential iron reducers is likely because (I) iron reducers were more efficient in the uptake of electron donors such as the provided acetate [122]; (II) iron reduction was the dominant biogeochemical process observed (as discussed above, Fig. 6B and S8A); or/and (III) sulfate reducers were thriving on different, sediment endogenous electron donors [123]. Nevertheless, the detection of *dsrA* transcripts in the SIP fractions (Fig. 6C) supported the hypothesis of co-occurring sulfate reduction in the treatments. Following the observations of the SIP experiment, we suggest that minor concurrent sulfate reduction, in the background of the dominant TEAP i.e. iron reduction, likely fuels the persistence of sulfate reducers in situ in the iron reduction sites Annenkov Trough, Cumberland Bay, and Drygalski Trough (Figs. 3A and 5). The non-detection of sulfide in the incubations could be explained by precipitation with Fe^2+^ forming mackinawite (FeS) and/or pyrite (FeS_2_) [124, 125], or reoxidation microbially or abiotically by reactive Fe(III) oxides [21]. Recently, this type of cryptic sulfur cycling,  indicated by high sulfide oxidation rates in surface sediments, was shown in multiple studies [21, 126, 127]. In addition, concurrent sulfate and iron reduction in the same zone was reported multiple times [17, 20, 77, 124], but detailed information about the associated microbial community is lacking. These studies [21, 126–129] assign the majority of ferrous iron production to the abiotic process of iron reduction by sulfide oxidation. Although this process likely also occurs in the sediments investigated in this study, sulfide concentrations below detection limit (Fig. 2) and the high activity of mainly iron reducing microorganisms (Fig. 6B) indicate that these abiotic processes provide a minor contribution to observed high Fe^2+^ concentrations (Figs. 2 and S8A).

A limitation of our study is the unexpected lack of dissolved Fe^2+^ over time in the acetate, lepidocrocite and molybdate treatment from the SIP incubation (Fig. S8A). While this result suggested that iron reduction did not occur in this treatment, the enrichment of *Desulfuromonadales* members in similar proportion as in the other treatments shows that iron reduction certainly occurred (Fig. 6B). Besides, inhibition of iron reduction by molybdate has not been shown previously. In comparable studies, molybdate concentrations of 10 mM [83] or even 20 mM [124] did not inhibit microbial iron reduction. Instead, in our study, the produced dissolved Fe^2+^ reacted abiotically with the added molybdate, preventing the detection of Fe^2+^ (see supplementary material, Figs. S11 and S12, for details).

## Conclusion

This study has shown how the microbial communities in sub-Antarctic South Georgia surface sediments are shaped by the dominant TEAP; sulfate reduction in Church Trough and iron reduction in Cumberland Bay, Drygalski Trough, and Annenkov Trough. We provide evidence for microbial iron reduction as one of the metabolic capabilities of the family Sva1033 using RNA-SIP with Cumberland Bay surface sediments. Coincidentally, in all iron reduction sites, Sva1033 was the dominant member of *Desulfuromonadales* found in situ, while other known marine iron reducers were scarce. We also identified iron-reducing capabilities of Sva1033 members in similar surface sediments from Potter Cove in the Antarctic Peninsula. Therefore, this clade might be very important for iron reduction in permanently cold marine sediments given the input of iron from enhanced glacial erosion, weathering, and glacial melt as a result of global warming. Furthermore, our data show high relative abundance of persistent sulfate reducers and suggest their activity in the iron reduction zone of marine sediments potentially participating in cryptic sulfur cycling, with the produced sulfide precipitating as metal sulfide mineral or being reoxidized.

## Supplementary information

Supplemental Material

## Data Availability

The raw sequence data of this study were submitted to GenBank Short Reads Archive (SRA) under the BioProject numbers PRJNA658241 and PRJNA668691 (BioSample numbers SAMN16419039–SAMN16419043 and SAMN16419024–SAMN16419028). The environmental geochemical data were submitted to PANGAEA data publisher for Earth & Environmental Science database under the following doi: 10.1594/PANGAEA.927101.
