# Supplementary material for: Iron and sulfate reduction structure microbial communities in (sub-)Antarctic sediments
Source: ISME J. 2021 Jun 21;15(12):3587–604. doi: 10.1038/s41396-021-01014-9 (PMC8630232; doi:10.1038/s41396-021-01014-9)
Supplement: Supplementary file 1 — Supplemental Material [file 41396_2021_1014_MOESM1_ESM.pdf]

**Iron and sulfate reduction structure microbial communities in (sub-)Antarctic sediments**

Running title: Iron and sulfate reduction in South Georgia sediments

Lea C. Wunder[1,2§], David A. Aromokeye[1,3§*], Xiuran Yin[1,3], Tim Richter-Heitmann[1], Graciana Willis-Poratti[1,4,5], Annika Schnakenberg[1,2], Carolin Otersen[1], Ingrid Dohrmann[6], Miriam Römer[3.7], Gerhard Bohrmann[3,7], Sabine Kasten[3,6,7] and Michael W. Friedrich[1,3*]

[1] Microbial Ecophysiology Group, Faculty of Biology/Chemistry, University of Bremen, Bremen, Germany

[2] Max Planck Institute for Marine Microbiology, Bremen, Germany

[3] MARUM – Center for Marine Environmental Sciences, University of Bremen, Bremen, Germany

[4] Instituto Antártico Argentino, 25 de Mayo 1143, 1650, San Martín, Buenos Aires, Argentina

[5] Facultad de Ciencias Exactas, Universidad Nacional de La Plata, Calle 115 y 47, 1900, La Plata, Buenos Aires, Argentina

[6] Alfred Wegener Institute Helmholtz Centre for Polar and Marine Research, Bremerhaven, Germany

[7] Faculty of Geosciences, University of Bremen, Bremen, Germany

§ These authors contributed equally to this work

* Corresponding author

Correspondence:

David A. Aromokeye

Michael W. Friedrich,

Microbial Ecophysiology Group, Faculty of Biology/Chemistry, University of Bremen, PO Box 33 04 40, D-28334 Bremen, Germany

Emails: david.aromokeye@uni-bremen.de; michael.friedrich@uni-bremen.de

# Contents

Supplementary figures ........................................................ 3

Supplementary tables ........................................................ 13

Supplementary material and methods ........................................................ 17

Experimental set up for stable isotope probing incubations ........................................................ 17

Sequencing analysis ........................................................ 17

$Fe^{2+}$ measurement in molybdate treated incubations – abiotic controls ........................................................ 18

References ........................................................ 20

## Supplementary figures

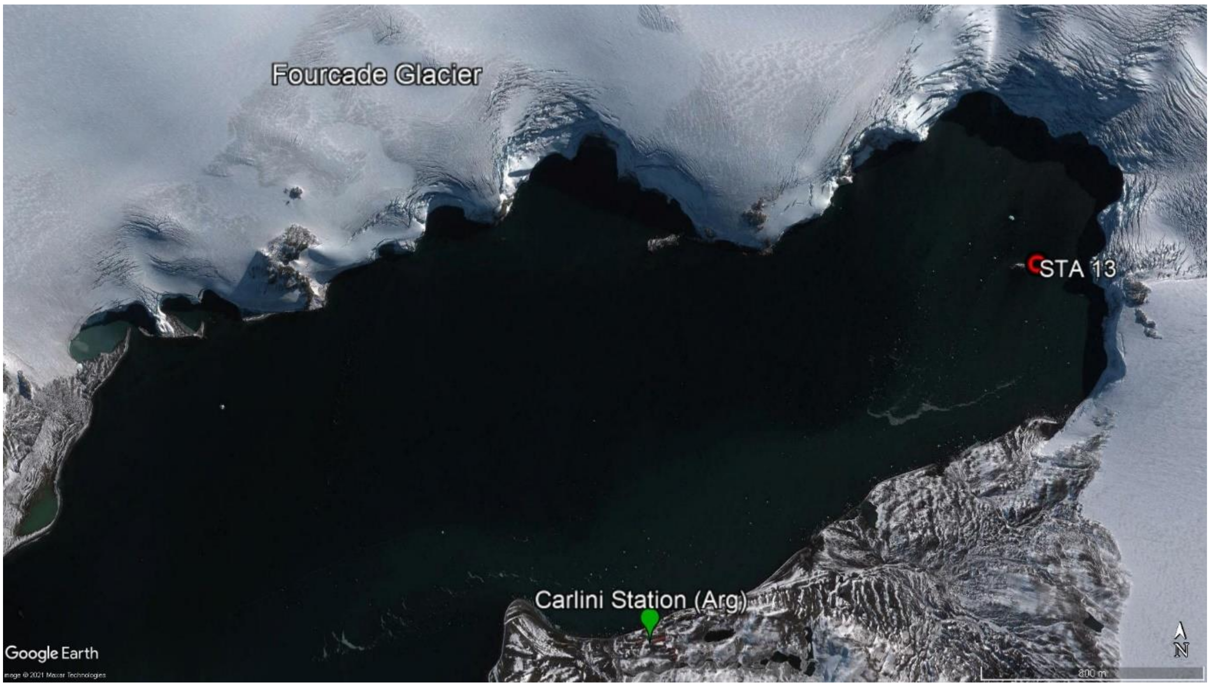


Fig. S1: Sampling location of Station 13 in Potter Cove (King George Island/Isla 25 de Mayo, Antarctic Peninsula). Produced with Google Earth.

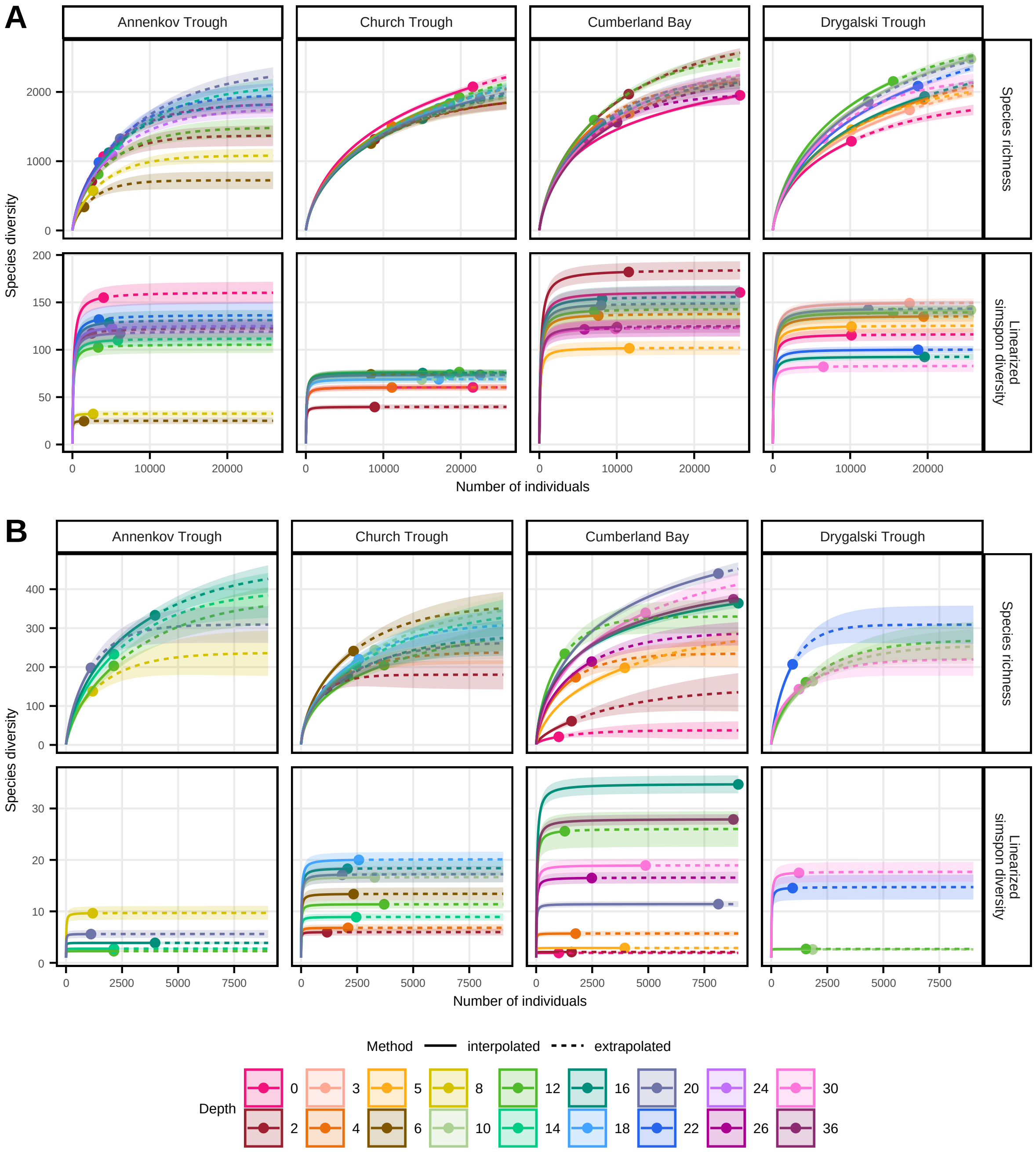


Fig. S2: Rarefaction curves of 16S rRNA sequencing of South Georgia surface sediment for bacteria (**a**) and archaea (**b**). Depth in cm below seafloor. **b** Samples after removing low read samples (see Table S2) leaving Annenkov Trough n = 5, Church Trough n = 9, Cumberland Bay n = 10, Drygalski Trough n = 4.

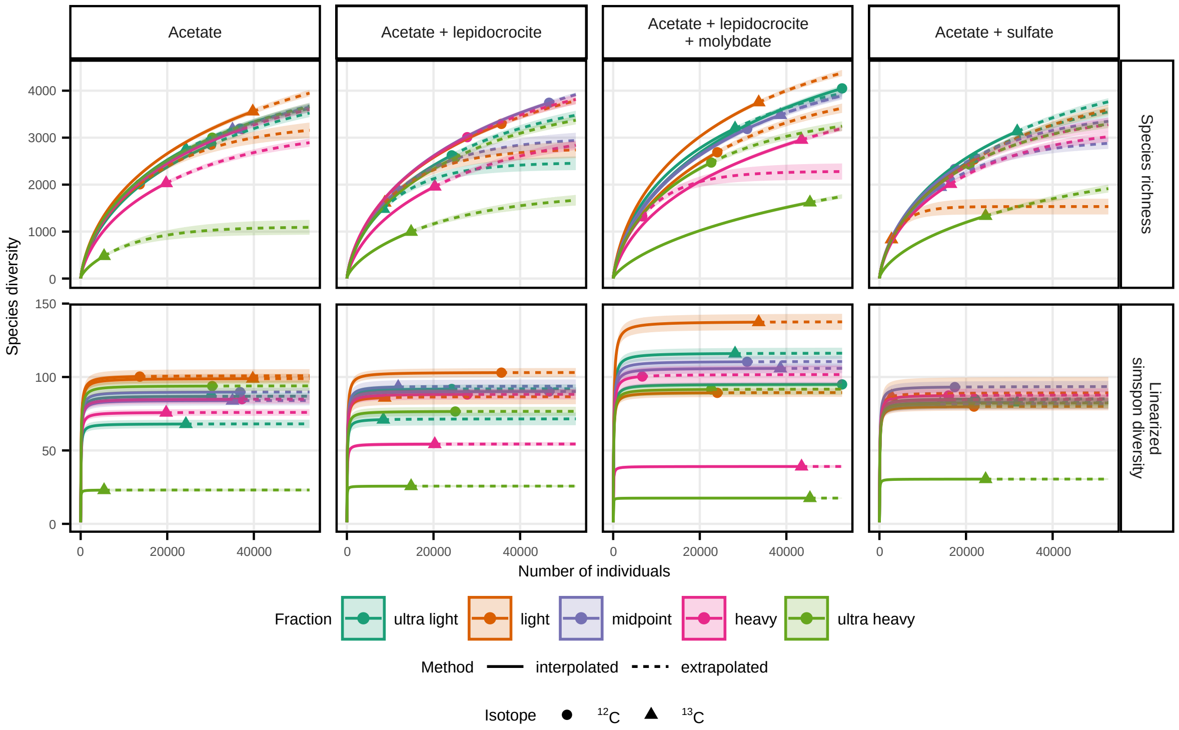


Fig. S3: Rarefaction curve of bacterial 16S rRNA sequencing of SIP incubations with Cumberland Bay sediments.

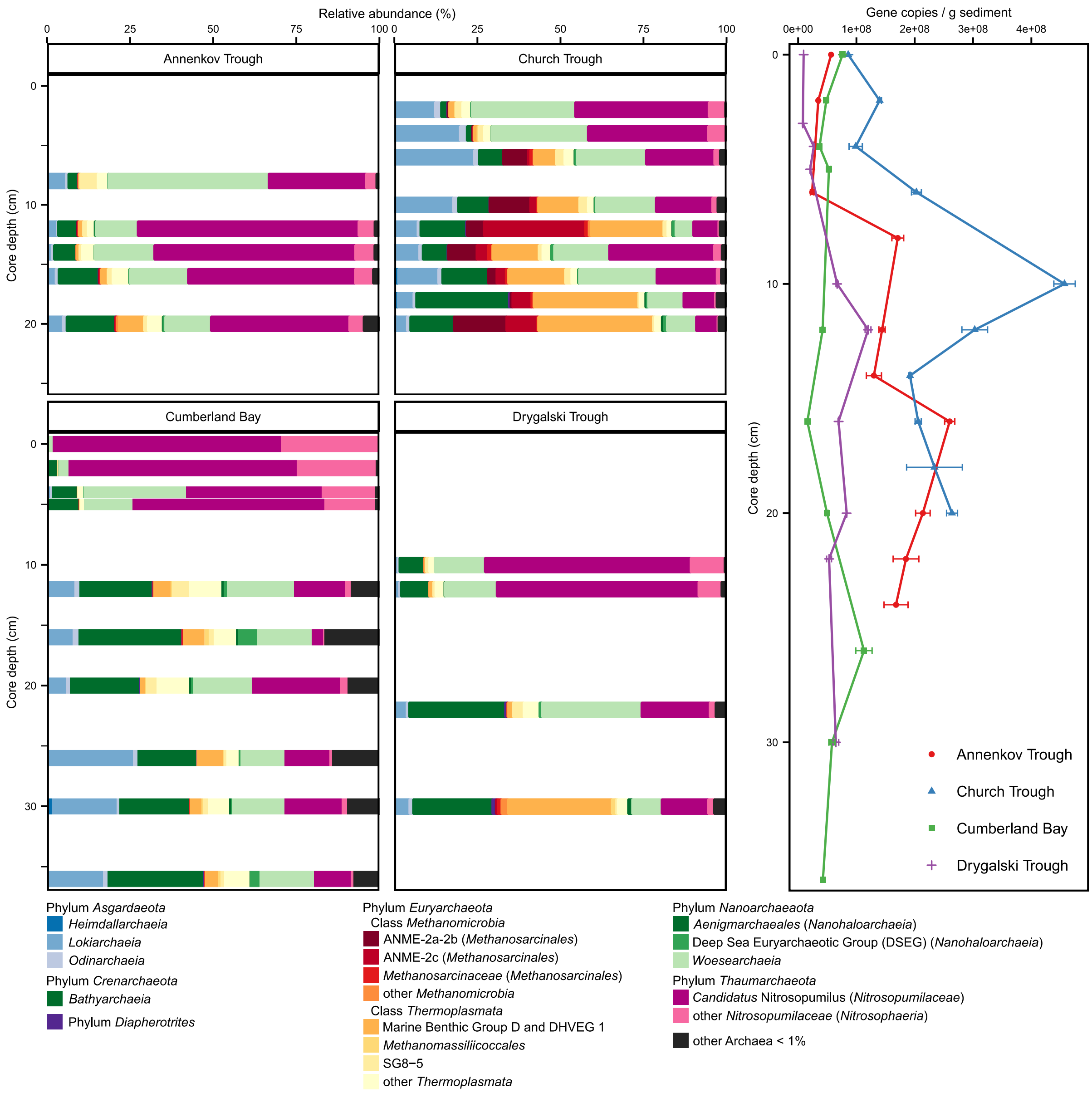


Fig. S4: Archaeal community composition and gene copy numbers in South Georgia surface sediments. **a** Relative abundance of bacterial 16S rRNA genes in Annenkov Trough, Church Trough, Cumberland Bay and Drygalski Trough. From the originally sequenced 10 samples per site, some were removed due to insufficient coverage (see Table S2). **b** Archaeal 16S rRNA gene copies per gram wet sediment of 10 samples per site with error bars displaying SD of technical qPCR replicates (n = 3).

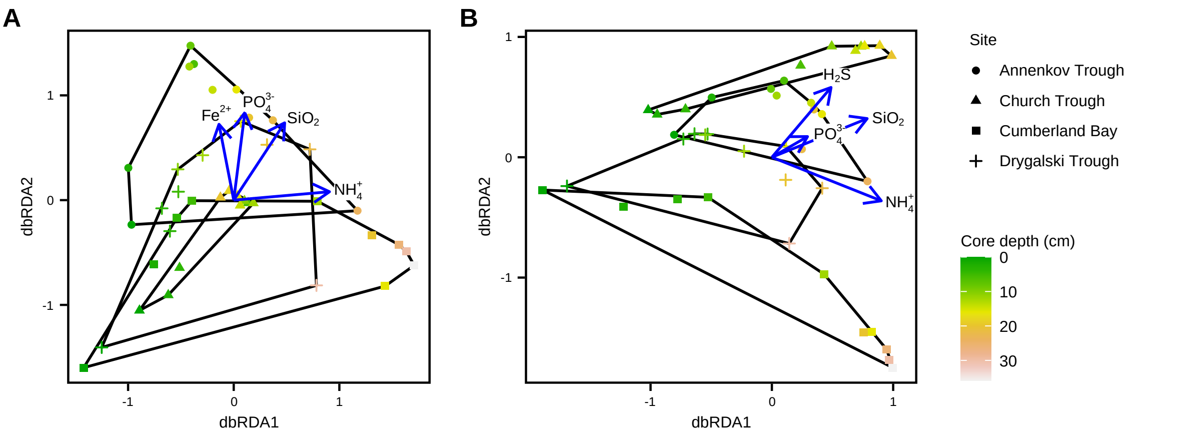


Fig. S5: Partial distance-based redundancy analysis (dbRDA) ordination plot of bacterial communities in surface sediments of South Georgia. Variation explained by (**a**) $H_2S$ or (**b**) $Fe^{2+}$ were removed from the model. Sample points are distinguished by site and core depth by shape and color respectively. dbRDA1 and dbRDA2 axes are displayed which constrain the Bray Curtis distance matrix with geochemical parameters $PO_4^{3-}$, $NH_4^+$, $SiO_2$ and $Fe^{2+}$ or $H_2S$. The total model (**a** $F = 4.13$, $p < .01$, Df 4, 34; **b** $F = 4.57$, $p < .01$, Df 4, 34) and each individual parameter ($p < 0.05$) was significant.

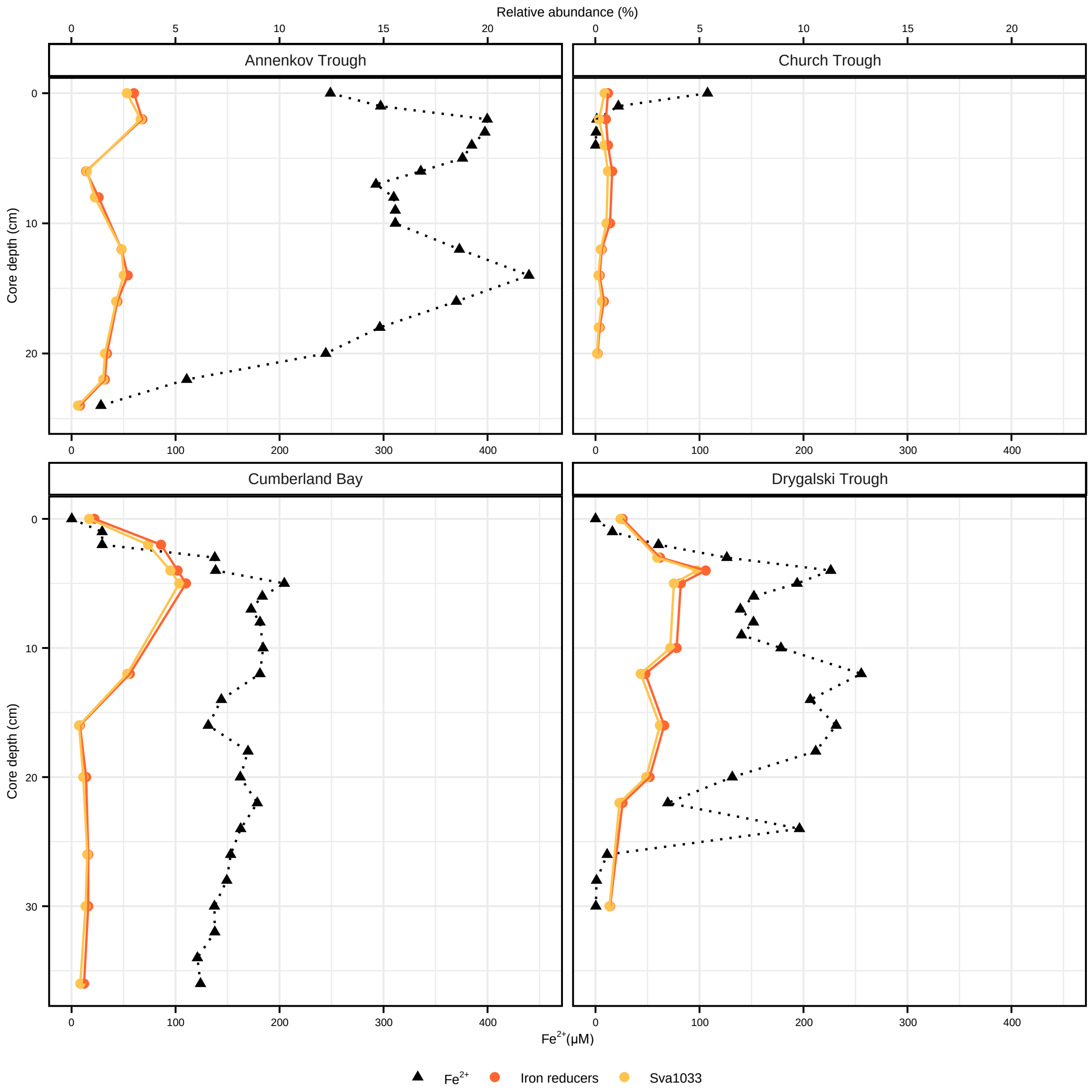


Fig. S6: Depth profile of contribution of iron reducing microorganisms in *Deltaproteobacteria* and family Sva1033 to bacterial 16S rRNA gene community in South Georgia surface sediments. $Fe^{2+}$ profile from Fig. 2 was displayed.

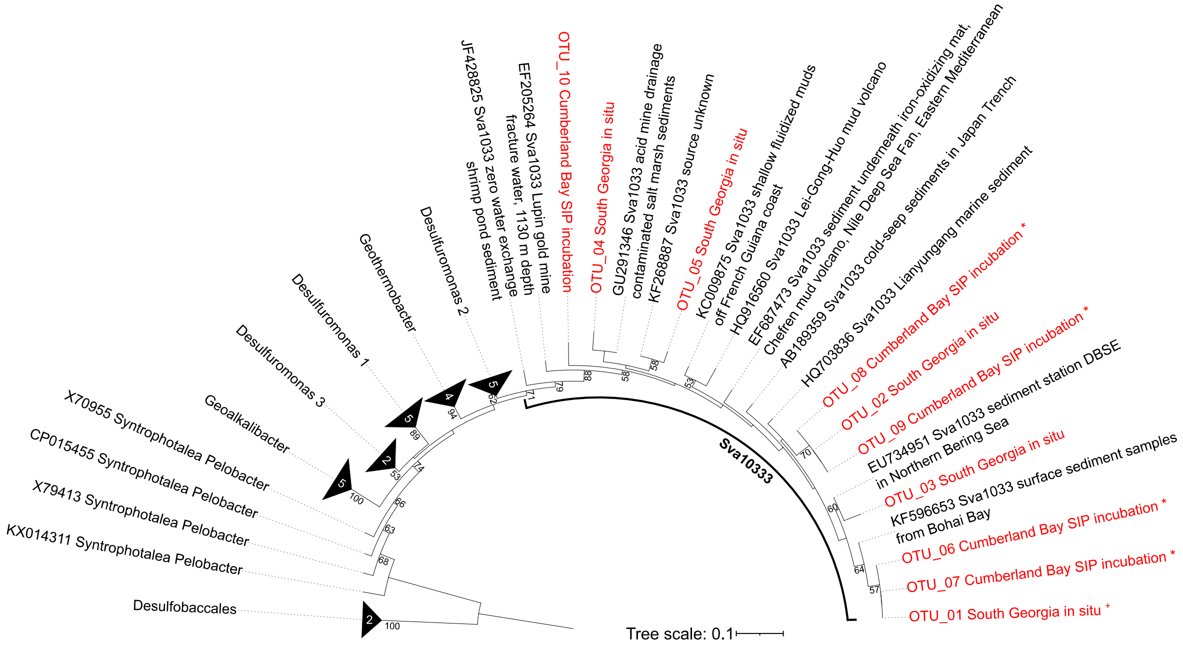


Fig. S7: Phylogenetic tree of family Sva1033 and closest sister clusters including the most abundant OTUs of this study (in red). Bootstrap values > 50% are shown in the tree. The reference sequences were exported from the ARB tree of SILVA release 138, Quast *et al.* (1). Accession numbers of sequences in collapsed nodes: Desulfobaccales FJ437876, AF002671; Geoalkalibacter CP010311, KJ817771, KT699114, DQ309326, MG602814; Desulfuromonas 3 JQ801020, JF727697; Desulfuromonas 1 JX223285, MF806540, JX224539, JX222942, HM141856; Geothermobacter KF741402, AY155599, GQ433952; Desulfuromonas 2 EU052234, KC470887, JX391250, KC471166, KM203496.

* : most abundant OTUs from SIP incubations representing together 96% of all Sva1033 sequences

$^{+}$ : most abundant OTU from *in situ* sediments representing 90% of all Sva1033 sequences

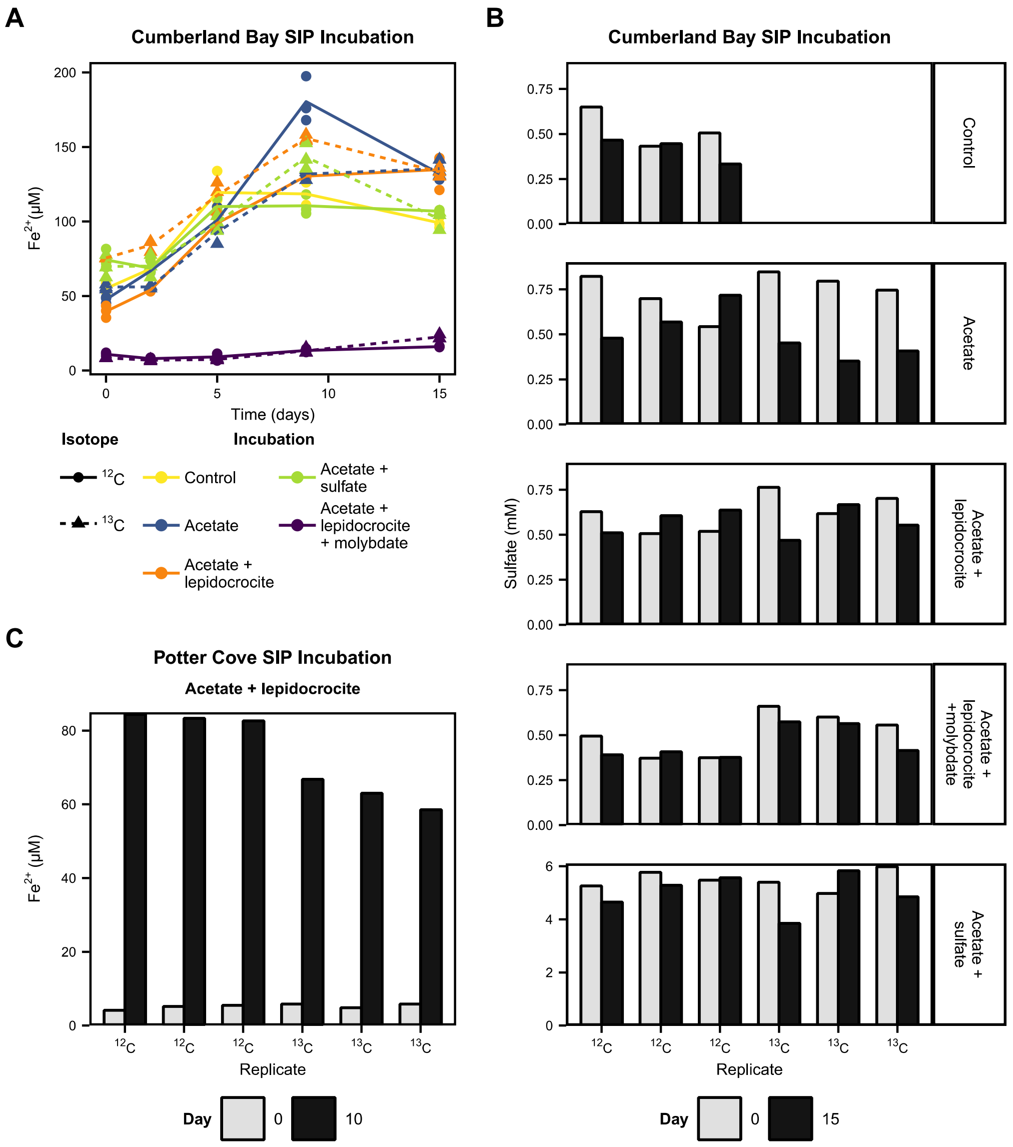


Fig. S8: Time course of $Fe^{2+}$ and sulfate concentrations in SIP incubations of Cumberland Bay and Potter Cove sediments. **a** $Fe^{2+}$ concentration of Cumberland Bay SIP incubations over time separated by treatment. Lines connect mean of triplicates of each treatment, separate for $^{12}C$ and $^{13}C$ acetate. **b** Sulfate concentration of Cumberland Bay SIP incubations of each replicate at start and end time point (day 0 – 15). The technical measurement error for sulfate measurements was 2%. **c** $Fe^{2+}$ concentration of single Potter Cove SIP incubation treatment at start and end time point (day 0 – 10).

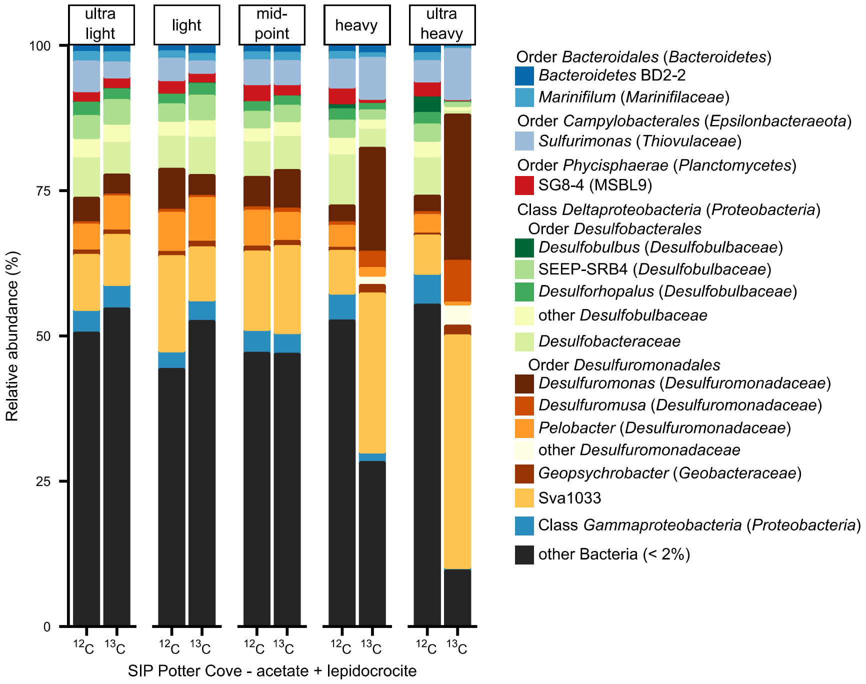


Fig. S9: SIP incubation of Potter Cove sediments. Density separated bacterial 16S rRNA community composition of taxa with > 2% relative abundance.

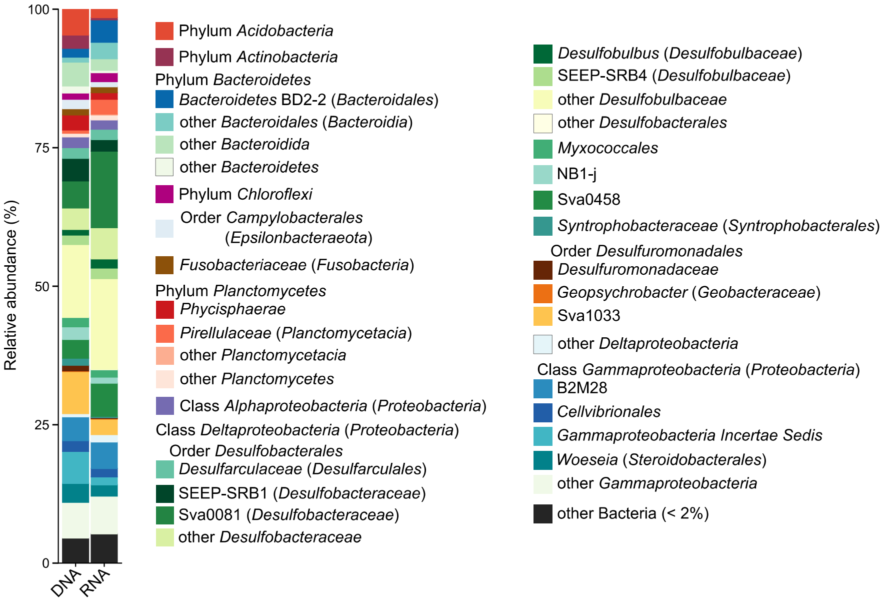


Fig. S10: SIP incubation of Cumberland Bay sediments bacterial 16S rRNA starting community on RNA and DNA level.

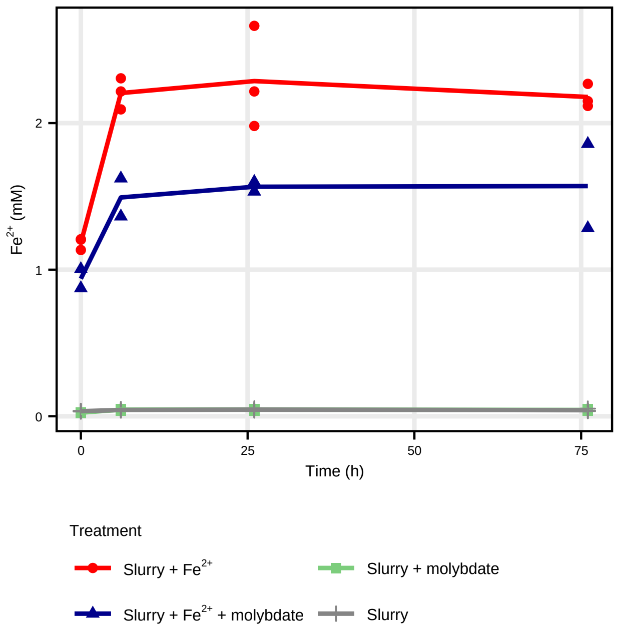


Fig. S11: Measured $Fe^{2+}$ of abiotic ferrous iron. The line connects the mean for replicates of each treatment with n = 3 for all except Slurry + $Fe^{2+}$ + molybdate with n = 2. For details see text below.

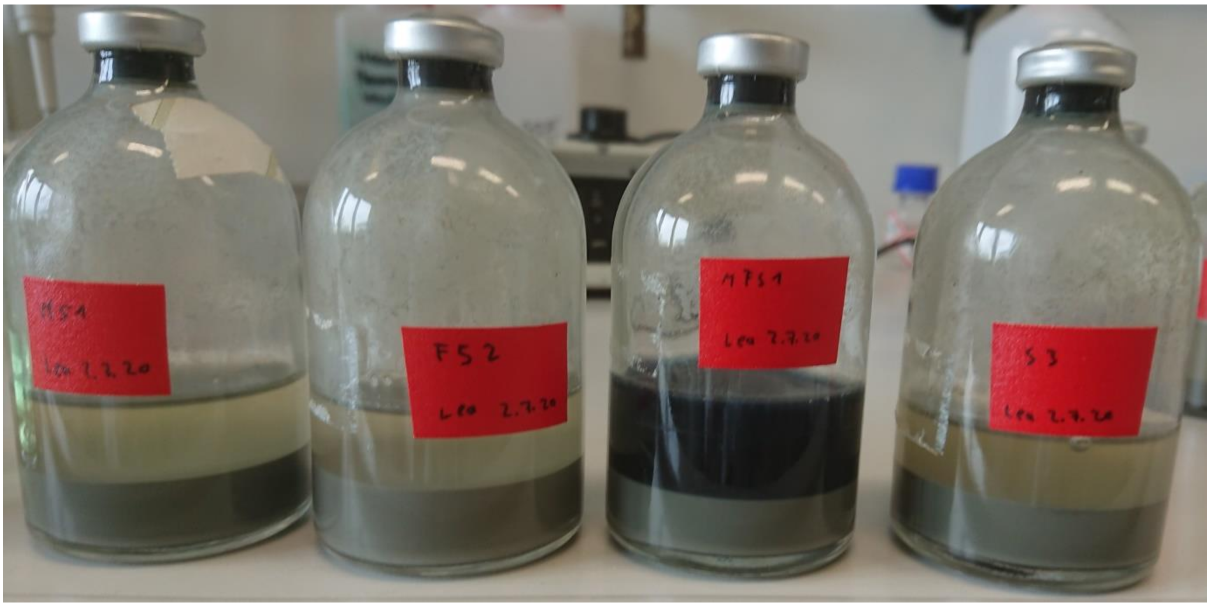

Fig. S12: Serum bottles of abiotic control experiment. One replicate of each treatment, from left to right: slurry + molybdate, slurry + $Fe^{2+}$, slurry + $Fe^{2+}$ + molybdate, slurry only.

# Supplementary tables

Table S1: Sampling location and sample details

| **Sampling objective** | **Site**<br>**Core ID** | **Core depth (cm)*** | **Coordinates** | |
|---|---|---|---|---|
| | | | **Latitude** | **Longitude** |
| Archaeal and bacterial community composition analysis and quantification, *dsrA* gene quantification | Annenkov Trough<br>GeoB22054-2<br>(MUC-12) | 0 – 1<br>2 – 3<br>6 – 7<br>8 – 9<br>12 – 14<br>14 – 16<br>16 – 18<br>20 – 22<br>22 – 24<br>24 – 26 | 54°26.169 S | 37°21.094 W |
| Geochemical measurements on pore water | | 0 – 10 every 1 cm<br>10 – 34 every 2 cm | | |
| Archaeal and bacterial community composition analysis and quantification, *dsrA* gene quantification | Church Trough<br>GeoB22031-1<br>(MUC-5) | 0 – 1<br>2 – 3<br>4 – 5<br>6 – 7<br>10 – 12<br>12 – 14<br>14 – 16<br>16 – 18<br>18 – 20<br>20 – 22 | 53°46.209 S | 38°08.413 W |
| Geochemical measurements on pore water | | 0 – 10 every 1 cm<br>10 – 30 every 2 cm | | |
| Archaeal and bacterial community composition analysis and quantification, *dsrA* gene quantification | Cumberland Bay<br>GeoB22046-1<br>(MUC-8) | 0 – 1<br>2 – 3<br>4 – 5<br>5 – 6<br>12 – 14<br>16 – 18<br>20 – 22<br>26 – 28<br>30 – 32<br>36 – 38 | 54°17.270 S | 36°27.710 W |
| Geochemical measurements on pore water | | 0 – 10 every 1 cm<br>10 – 40 every 2 cm | | |
| Archaeal and bacterial community composition analysis and quantification, *dsrA* gene quantification | Drygalski Trough<br>GeoB22015-1<br>(MUC-4) | 0 – 1<br>3 – 4<br>4 – 5<br>5 – 6<br>10 – 12<br>12 – 14<br>16 – 18<br>20 – 22<br>22 – 24<br>30 – 32 | 54°51.269 S | 35°54.667 W |
| Geochemical measurements on pore water | | 0 – 10 every 1 cm<br>10 – 32 every 2 cm | | |
| SIP incubation | Cumberland Bay<br>GeoB22024-1 (GC-6) | 0 – 14 | 54°15.885 S | 36°26.225 W |
| SIP incubation | Potter Cove<br>Station 13-04 | 0 – 29<br>(whole core) | 62°13.523 S | 58°38.470 W |

* In the text, other tables and figures only start depth is displayed

Table S2: Sequencing details surface sediments, South Georgia
Red labelled archaea samples were removed from the analyses due to insufficient sequencing depth

| Site<br>Core ID | Core depth (cm) | Total reads | | Total OTUs | |
|---|---|---|---|---|---|
| | | Bacteria | Archaea | Bacteria | Archaea |
| Annenkov Trough<br>GeoB22054-2 | 0 | 4025 | 423 | 1067 | 74 |
| | 2 | 2497 | 334 | 707 | 80 |
| | 6 | 1494 | 670 | 340 | 133 |
| | 8 | 2680 | 1192 | 573 | 140 |
| | 12 | 3326 | 2132 | 814 | 204 |
| | 14 | 5870 | 2138 | 1236 | 235 |
| | 16 | 4712 | 3973 | 1123 | 336 |
| | 20 | 6165 | 1113 | 1325 | 199 |
| | 22 | 3420 | 502 | 982 | 121 |
| | 24 | 5129 | 618 | 1093 | 133 |
| Church Trough<br>GeoB22031-1 | 0 | 21567 | 761 | 2074 | 111 |
| | 2 | 8887 | 1167 | 1316 | 146 |
| | 4 | 11103 | 2093 | 1504 | 182 |
| | 6 | 8405 | 2342 | 1255 | 243 |
| | 10 | 14952 | 3284 | 1607 | 246 |
| | 12 | 19810 | 3706 | 1923 | 206 |
| | 14 | 18582 | 2456 | 1833 | 207 |
| | 16 | 15080 | 2071 | 1616 | 177 |
| | 18 | 17165 | 2579 | 1764 | 222 |
| | 20 | 22506 | 1829 | 1898 | 170 |
| Cumberland Bay<br>GeoB22046-1 | 0 | 25910 | 1009 | 1951 | 21 |
| | 2 | 11546 | 1581 | 1968 | 61 |
| | 4 | 7618 | 1761 | 1548 | 174 |
| | 5 | 11630 | 3952 | 1693 | 198 |
| | 12 | 7098 | 1280 | 1594 | 235 |
| | 16 | 8106 | 9021 | 1516 | 367 |
| | 20 | 7996 | 8132 | 1546 | 442 |
| | 26 | 5828 | 2481 | 1263 | 214 |
| | 30 | 9828 | 4883 | 1616 | 341 |
| | 36 | 10034 | 8799 | 1562 | 375 |
| Drygalski Trough<br>GeoB22015-1 | 0 | 10154 | 122 | 1288 | 37 |
| | 3 | 17665 | 142 | 1740 | 41 |
| | 4 | 19482 | 568 | 1892 | 90 |
| | 5 | 10165 | 805 | 1455 | 103 |
| | 10 | 25580 | 1853 | 2476 | 166 |
| | 12 | 15554 | 1551 | 2151 | 161 |
| | 16 | 19605 | 732 | 1936 | 129 |
| | 20 | 12337 | 451 | 1859 | 137 |
| | 22 | 18759 | 955 | 2084 | 207 |
| | 30 | 6532 | 1239 | 1328 | 144 |

Table S3: SIP incubation set-up Cumberland Bay

| Treatment (n = 3) | Acetate (500 µM) | Lepidocrocite (5 mM) | Sulfate (5 mM) | Molybdate (10 mM) | Days pre-incubation |
|---|---|---|---|---|---|
| Control | | | | | 4 |
| Acetate | $^{12}C$ | | | | 6 |
| Acetate | $^{13}C$ | | | | 6 |
| Acetate + lepidocrocite | $^{12}C$ | x | | | 6 |
| Acetate + lepidocrocite | $^{13}C$ | x | | | 6 |
| Acetate + sulfate | $^{12}C$ | | x | | 6 |
| Acetate + sulfate | $^{13}C$ | | x | | 6 |
| Acetate + lepidocrocite + molybdate | $^{12}C$ | x | | x | 4 |
| Acetate + lepidocrocite + molybdate | $^{13}C$ | x | | x | 6 |

Table S4.1: Sequencing details SIP incubation samples

| Treatment | Isotope | Fraction | Total reads | Total OTUs | Density (g/ml) |
|---|---|---|---|---|---|
| Cumberland Bay Acetate | $^{12}C$ | 3+4 | 30386 | 3000 | 1.818 – 1.826 |
| | | 5+6 | 37248 | 3184 | 1.803 – 1.810 |
| | | 7+8 | 36816 | 3194 | 1.791 – 1.799 |
| | | 9+10 | 13656 | 2006 | 1.776 – 1.783 |
| | | 11+12 | 30136 | 2849 | 1.760 – 1.768 |
| | $^{13}C$ | 3+4 | 5410 | 479 | 1.814 – 1.822 |
| | | 5+6 | 19743 | 2030 | 1.799 – 1.806 |
| | | 7+8 | 35068 | 3172 | 1.783 – 1.791 |
| | | 9+10 | 39751 | 3557 | 1.768 – 1.776 |
| | | 11+12 | 24305 | 2753 | 1.753 – 1.760 |
| Cumberland Bay Acetate + lepidocrocite | $^{12}C$ | 3+4 | 25033 | 2575 | 1.814 – 1.822 |
| | | 5+6 | 27784 | 3010 | 1.799 – 1.806 |
| | | 7+8 | 46695 | 3743 | 1.787 – 1.791 |
| | | 9+10 | 35683 | 3293 | 1.772 – 1.779 |
| | | 11+12 | 24208 | 2623 | 1.756 – 1.764 |
| | $^{13}C$ | 3+4 | 14822 | 995 | 1.814 – 1.818 |
| | | 5+6 | 20291 | 1958 | 1.799 – 1.806 |
| | | 7+8 | 11846 | 1886 | 1.783 – 1.791 |
| | | 9+10 | 8732 | 1616 | 1.768 – 1.776 |
| | | 11+12 | 8416 | 1483 | 1.753 – 1.760 |
| Cumberland Bay Acetate + sulfate | $^{12}C$ | 3+4 | 20815 | 2427 | 1.818 – 1.822 |
| | | 5+6 | 15954 | 2151 | 1.799 – 1.806 |
| | | 7+8 | 17408 | 2333 | 1.783 – 1.791 |
| | | 9+10 | 21839 | 2534 | 1.772 – 1.776 |
| | | 11+12 | 22078 | 2570 | 1.756 – 1.764 |
| | $^{13}C$ | 3+4 | 24520 | 1333 | 1.814 – 1.822 |
| | | 5+6 | 16403 | 2014 | 1.799 – 1.806 |
| | | 7+8 | 14038 | 1950 | 1.783 – 1.791 |
| | | 9+10 | 2782 | 834 | 1.768 – 1.776 |
| | | 11+12 | 31827 | 3133 | 1.753 – 1.760 |

Table S4.2: Sequencing details SIP incubation samples

| Treatment | Isotope | Fraction | Total reads | Total OTUs | Density (g/ml) |
|---|---|---|---|---|---|
| Cumberland Bay Acetate + lepidocrocite + molybdate | $^{12}C$ | 3+4 | 22646 | 2470 | 1.814 – 1.820 |
| | | 5+6 | 6721 | 1324 | 1.799 – 1.806 |
| | | 7+8 | 30945 | 3186 | 1.783 – 1.791 |
| | | 9+10 | 24045 | 2689 | 1.768 – 1.776 |
| | | 11+12 | 52866 | 4049 | 1.756 – 1.760 |
| | $^{13}C$ | 3+4 | 45440 | 1620 | 1.814 – 1.818 |
| | | 5+6 | 43504 | 2954 | 1.799 – 1.806 |
| | | 7+8 | 38599 | 3482 | 1.783 – 1.791 |
| | | 9+10 | 33612 | 3752 | 1.768 – 1.776 |
| | | 11+12 | 28149 | 3196 | 1.756 – 1.764 |
| Potter Cove Acetate + lepidocrocite | $^{12}C$ | 3+4 | 45543 | 3806 | 1.815 – 1.817 |
| | | 5+6 | 44232 | 3689 | 1.803 – 1.806 |
| | | 7+8 | 53991 | 3738 | 1.792 – 1.794 |
| | | 9+10 | 54971 | 3609 | 1.780 – 1.783 |
| | | 11+12 | 26062 | 2756 | 1.769 – 1.774 |
| | $^{13}C$ | 3+4 | 10703 | 629 | 1.820 – 1.826 |
| | | 5+6 | 2123 | 476 | 1.809 – 1.815 |
| | | 7+8 | 31702 | 3045 | 1.797 – 1.803 |
| | | 9+10 | 15678 | 2245 | 1.789 – 1.794 |
| | | 11+12 | 20690 | 2647 | 1.783 – 1.777 |

Table S5: Primer details 16S rRNA gene qPCR

| Primer | Sequence (5'-3') | Target | Denaturation time | Reference |
|---|---|---|---|---|
| Bac8Fmod | AGAGTTTGATYMTGGCTCAG | bacteria | 15 s | modified from (2) |
| Bac338Rmod | GCWGCCWCCCGTAGGWGT | bacteria | 15 s | modified from (3) |
| 27F | AGAGTTTGATCCTGGCTCAG | bacteria | | (4) |
| Ba1492 | GGTTACCTTGTTACGACTT | bacteria | | (4) |
| Ar806F* | ATTAGATACCCSBGTAGTCC | archaea | 30 s | (3) |
| Ar912rt | GTGCTCCCCCGCCAATTCCTTTA | archaea | 30 s | (5) |
| Ar109F | ACKGCTCAGTAACACGT | archaea | | (6) |
| A1492 | GGCTACCTTGTTACGACTT | archaea | | (4) |

* alternative name Arc787F

## Supplementary material and methods

### Experimental set up for stable isotope probing incubations

Anoxic slurries were prepared by homogenizing sediment with sulfate-free artificial sea water (per liter 26.4 g NaCl, 11.2 g $MgCl_2 \cdot 6 H_2O$, 1.5 g $CaCl_2 \cdot 2 H_2O$, 0.7 g KCl, prepared with purified water (Milli-Q)) at a ratio of 1:4 under a stream of nitrogen gas ($N_2$ 5.0). 40 ml slurry was transferred into 120 ml serum bottles sealed with butyl rubber stoppers. The headspace gas was exchanged with $N_2$. The detailed set up is shown in Table S3. Both C-atoms in acetate were $^{13}$C-labelled. Incubation was conducted at 5°C in the dark for a total of 15 days after substrate addition.

When samples for $Fe^{2+}$ measurements were taken anoxically, 1 ml slurry was frozen for later analyses. These samples were subsequently used to determine aqueous sulfate concentrations by fixing 200 µl slurry supernatant in 800 µl 1% zinc acetate. The measurement was performed with a Metrohm 930 Compact IC Flex ion chromatograph (sulfate detection limit 50 µM).

For RNA extraction the slurry of treatment triplicates were pooled and 15 ml were used in order to retrieve sufficient biomass for fractionation.

### Sequencing analysis

The sequence read analysis of surface sediment samples was performed as previously described (7) with updated software, using the QIIME 1.9.0 and USEARCH 11.0. For sequencing data of the *in situ* surface sediment samples (2x 150 bp), only the forward reads were used for further analysis and truncated to a minimum sequence length of 143 bp.

For sequences of SIP incubation samples (2x 250 bp), the pipeline was modified in its first steps before de-replication: forward and reverse reads were joined with minimum overlap of 10 bases followed by de-multiplexing and quality filtering to minimum sequence length of 242 bp and expected error of $< 0.5$ using QIIME 1.9.0 and USEARCH 11.0. The taxonomic assignment was based on the 16S rRNA database Silva release 132 (1).

Unassigned reads or assigned as archaea, chloroplast or mitochondria were removed from the bacterial OTU tables and respectively bacterial and unassigned reads were removed from the archaeal OTU table prior to further analyses. Sequencing details are provided in Table S2 and S4. Rarefaction curves were generated (vegan package (8)) and all samples not reaching the inflection point of the rarefaction curve were removed from the dataset, as their community coverage was considered insufficient (Fig. S2, S3). Differing sample sizes were normalized by scaling OTU abundance to the observation totals in each sample ("relative data"). Separately, the relative abundance of each taxon on all available ranks was summed up, i.e. for all phyla, classes, orders and so on.

## $Fe^{2+}$ measurement in molybdate treated incubations – abiotic controls

During the course of the stable isotope probing (SIP) incubations with Cumberland Bay sediments, measured $Fe^{2+}$ concentrations in the treatment containing acetate + lepidocrocite + molybdate was much lower compared to the other incubations, including the control (Fig. S8). However, the microbial community from the sequencing results indicated on-going iron reduction: the same known iron reducing microorganisms as in the other incubations were present and active (Fig. 6). One hypothesis for the lower concentrations of detectable $Fe^{2+}$ concentrations was abiotic reaction of $Fe^{2+}$ from iron reduction with molybdate. To address this hypothesis, supplementary incubations were set up and the findings are discussed below.

**Material and methods**

Experiments investigating the abiotic reaction of $Fe^{2+}$ and molybdate were set up in 120 ml serum bottles with 40 ml 1:4 slurry containing 10 g Cumberland Bay sediment (gravity core, 0 – 14 cm, same as used for main SIP experiments) and 30 ml artificial sea water (ASW, see supplementary methods above and main text). The slurry was autoclaved and all oxygen removed by flushing with $N_2$ gas before the substrate was added. Four treatments were set-up

containing 10 mM molybdate (n = 3), 1 mM $Fe^{2+}$ (n = 3, added as $FeCl_2$), both together (n = 2) or only sediment (n = 3).

$Fe^{2+}$ measurements were performed following the ferrozine assay from Viollier *et al.* (9). $Fe^{2+}$ was measured the first time directly after the substrate was added to all treatments, followed by measurements after 6, 26 and 76 h (Fig. S11). During that time, the treatments were incubated at 5°C in the dark.

**Results and discussion**

The $Fe^{2+}$ concentrations showed clear differences between the treatments (Fig. S11). Lower $Fe^{2+}$ concentrations were measured in the treatment slurry + $Fe^{2+}$ + molybdate (7 – 13 mM) compared to the treatment slurry + $Fe^{2+}$ (11 – 19 mM). $Fe^{2+}$ concentrations in the other treatments slurry only and slurry + molybdate stayed very low between 0.025 – 0.038 mM and 0 – 0.006 mM respectively. An immediate color change was observed in the slurry + $Fe^{2+}$ + molybdate treatment after adding the substrates, but not in any of the other control treatments (Fig. S12). Higher $Fe^{2+}$ concentrations were observed than initial $Fe^{2+}$ was added in according incubations. The addition of $Fe^{2+}$ in the form of $FeCl_2$ lowered the pH in these treatments probably resulting in the elution of $Fe^{2+}$ from the sediment particles.

The observations from these abiotic sediment incubations give clear indication for an abiotic reaction between the added $Fe^{2+}$ and the molybdate, therefore limiting the possibility to measure the exact levels of iron reduction in the acetate, lepidocrocite and molybdate treatments of the initial experiments (Fig. S8A). In summary, based on the results from these abiotic sediment incubations, we argue that in the acetate, lepidocrocite and molybdate treatments (Fig. 6 main text, Fig. S8A), iron reduction was on-going but most of the $Fe^{2+}$ formed reacted abiotically with molybdate.

# References

1. Quast C, Pruesse E, Yilmaz P, Gerken J, Schweer T, Yarza P, *et al*. The SILVA ribosomal RNA gene database project: improved data processing and web-based tools. *Nucleic Acids Res* 2012; **41**: D590-D596.

2. Eden PA, Schmidt TM, Blakemore RP, Pace NR. Phylogenetic analysis of *Aquaspirillum magnetotacticum* using Polymerase Chain Reaction-amplified 16S rRNA-specific DNA. *Int J Syst Evol Microbiol* 1991; **41**: 324-325.

3. Yu Y, Lee C, Kim J, Hwang S. Group-specific primer and probe sets to detect methanogenic communities using quantitative real-time polymerase chain reaction. *Biotechnol Bioeng* 2005; **89**: 670-679.

4. Lane DJ. 16S/23S rRNA sequencing. In: Stackebrandt E, Goodfellow M (eds). *Nucleic acid techniques in bacterial systematics*. John Wiley and Sons: New York, 1991, pp 115-175.

5. Lueders T, Friedrich MW. Effects of amendment with ferrihydrite and gypsum on the structure and activity of methanogenic populations in rice field soil. *Appl Environ Microbiol* 2002; **68**: 2484-2494.

6. Großkopf R, Janssen PH, Liesack W. Diversity and structure of the methanogenic community in anoxic rice paddy soil microcosms as examined by cultivation and direct 16S rRNA gene sequence retrieval. *Appl Environ Microbiol* 1998; **64**: 960-969.

7. Aromokeye DA, Richter-Heitmann T, Oni OE, Kulkarni A, Yin X, Kasten S, *et al*. Temperature controls crystalline iron oxide utilization by microbial communities in methanic ferruginous marine sediment incubations. *Front Microbiol* 2018; **9**: 2574.

8. Oksanen J, Blanchet FG, Friendly M, Kindt R, Legendre P, McGlinn D, *et al*. vegan: Community Ecology Package, 2.5-6. 2019. Available from: https://CRAN.R-project.org/package=vegan

9. Viollier E, Inglett P, Hunter K, Roychoudhury A, Van Cappellen P. The ferrozine method revisited: Fe(II)/Fe(III) determination in natural waters. *Appl Geochem* 2000; **15**: 785-790.
